# Smart Technology Facilitated Patient-Centered Venous Thromboembolism Management (the SmaVTE Study): Protocol for a Randomized Controlled Trial

**DOI:** 10.2196/67254

**Published:** 2025-06-05

**Authors:** Zhi-Geng Jin, Zhe-Qi Zhang, Bin-Bin Liu, Hao Wang, Ying Yang, Li-Na Ren, Hui Zhang, Wei Ji, Zhen-Guo Zhai, Yu-Tao Guo

**Affiliations:** 1 Department of Pulmonary Vascular and Thrombotic Disease Sixth Medical Center of Chinese PLA General Hospital Beijing China; 2 Chinese PLA Medical School Beijing China; 3 Department of Cardiology Second Medical Center of Chinese PLA General Hospital Beijing China; 4 Quality Management Division Sixth Medical Center of Chinese PLA General Hospital Beijing China; 5 Department of Pulmonary and Critical Care Medicine Center of Respiratory Medicine China-Japan Friendship Hospital Beijing China

**Keywords:** venous thromboembolism, deep vein thrombosis, pulmonary embolism, prophylaxis, mobile health, patient-centered care, patient education, thrombosis, thrombus, mHealth, mobile applications, mobile apps, apps

## Abstract

**Background:**

Venous thromboembolism (VTE) is a significant public health issue, with a rising global incidence despite extensive research efforts. Patient-centered care, which tailors treatment to individual needs, has shown potential in enhancing outcomes. The integration of smart technologies with psychological frameworks such as the health belief model and the knowledge-attitude-practice (KAP) model may further improve patient engagement and adherence. To address this, we have developed a smart technique–assisted patient-centered care mobile health app for managing VTE (mVTEA), which integrates psychological frameworks to improve patient outcomes in VTE management.

**Objective:**

This study aims to investigate the impact of the mVTEA app on the knowledge, attitudes, and practices of VTE in patients with or at high risk of VTE.

**Methods:**

The SmaVTE (smart technology facilitated patient-centered venous thromboembolism management) study is a 2-armed, single-center, parallel-group, randomized controlled trial. A total of 256 hospitalized patients with or at high risk of VTE will be recruited on the day of their discharge from August 2024 to June 2025. Participants will be randomly allocated to either the mVTEA management group or the routine management group in a 1:1 ratio. The mVTEA management group (n=128) will receive patient-centered VTE management facilitated by the mVTEA app after discharge. The routine management group (n=128) will be administered conventional postdischarge management according to local clinical practice. The KAP of patients will be assessed by a structured KAP questionnaire on VTE. The primary outcome is the difference in patients’ KAP on VTE at 3-month follow-up between the 2 groups. Secondary outcomes include scores on each domain of the questionnaire, quality of life, VTE events, chronic thromboembolic pulmonary hypertension, chronic thromboembolic pulmonary disease, postpulmonary embolism syndrome, major bleeding events, VTE-related hospitalizations or rehospitalizations, deaths, and new-onset atrial fibrillation or atrial flutter at 3-month follow-up.

**Results:**

Participants are currently being recruited. The first participant was enrolled in August 2024, which marked the official start of the study. The recruitment process is expected to be completed in June 2025. As of the submission of the paper, 185 patients had been enrolled in this clinical trial. At present, all included patients are being followed up according to the outlined schedule.

**Conclusions:**

The SmaVTE study offers a pioneering approach to VTE prevention and treatment by combining smart technology with patient-centered care and established theoretical frameworks. The findings could significantly impact clinical practice and inspire further research into the integration of smart technologies with behavioral science theories.

**Trial Registration:**

ClinicalTrials.gov NCT06350331; https://clinicaltrials.gov/study/NCT06350331

**International Registered Report Identifier (IRRID):**

DERR1-10.2196/67254

## Introduction

### Background

Venous thromboembolism (VTE), which encompasses deep vein thrombosis and pulmonary embolism (PE), affects more than 1 in 12 individuals over their lifetime and poses a substantial health and economic burden [1-4]. Despite decades of research on the prevention and treatment of VTE, the prevalence of VTE has substantially increased worldwide [5-7]. Up to 75% of VTE cases occur in the hospital or within 90 days after hospital discharge [2,8], highlighting the complexity and challenge of VTE management.

Studies suggest that many prescribed pharmacologic prophylaxes are not administered due to patient refusal or insufficient patient-nurse communication [9-12]. Even among patients who commence anticoagulant therapy, many discontinue treatment after bleeding or other setbacks. Clinicians also face the difficult task of balancing thrombosis and bleeding risks, which often share overlapping risk factors that can change over time [13,14]. Determining an optimal, individualized decision is therefore fundamental to improving VTE management.

Patient-centered care, recommended by cardiovascular guidelines [15,16], emphasizes meeting patients’ unique needs and involving them in decision-making. Adoption of a patient-centered approach has demonstrated benefits in cardiovascular disease, improving patient knowledge, self-efficacy, and health outcomes [17,18]. A limited number of studies suggest similar benefits in VTE management [19-21], but further research is needed on the role of patient-centered care for patients with or at risk of VTE.

Smart technology, such as wearable devices, mobile health (mHealth) apps, artificial intelligence, and digital communication platforms, has shown potential in facilitating patient education, providing real-time feedback, and enhancing patient engagement [22-24]. The use of smart technology in patient-centered care supports personalized and proactive health care by empowering patients to manage their health, which is especially important for VTE, where patient behavior greatly affects outcomes [13,16,25]. A cohort study has shown that an mHealth-based web-based anticoagulation management system can effectively improve patient care and safety for warfarin-treated patients with VTE [26].

Although mHealth is effective, patient engagement tends to wane over time [27,28]. Furthermore, studies on health behavior suggest that simply emphasizing risk does not necessarily promote better adherence, due to psychological phenomena such as “optimistic bias” [29,30]. Various theoretical models have been advanced to explain health behaviors, with the health belief model (HBM) being one of the most common [31-34]. Studies demonstrate that HBM-based interventions can enhance self-management [33]; yet, their ability to predict health behaviors is limited [35]. The knowledge-attitudes-practices (KAP) model offers complementary insights, linking knowledge to behavioral attitudes, and thereby representing another potentially useful tool [32,34]. To the best of our knowledge, there is a lack of research in the field of VTE that integrates the theories of HBM and KAP, complemented by mHealth technology.

To address this research gap, we have recently developed a smart technology–assisted, patient-centered care mHealth app for managing VTE (mVTEA) [36]. Its goal is to improve patient-centered care for patients with or at risk of VTE by combining HBM and KAP frameworks.

### Objective

The SmaVTE (smart technology facilitated patient-centered venous thromboembolism management) study aims to investigate the impact of the mVTEA app on the KAP of patients with or at high risk of VTE assessed by a VTE-KAP questionnaire in a 3-month randomized controlled trial. We hypothesized that this theory-based mHealth intervention can improve the KAP of these patients while ensuring their safety compared to traditional face-to-face medical visits.

## Methods

### Study Design and Setting

The SmaVTE study is a 2-armed, single-center, parallel-group, randomized controlled trial designed to evaluate the impact of the mVTEA app on the KAP of patients with or at high risk of VTE. The trial will be conducted at the Sixth Medical Center of the Chinese People’s Liberation Army General Hospital, which is a tertiary hospital that integrates medical care, teaching, and research. A total of 256 hospitalized patients with or at high risk of VTE will be recruited on the day of their discharge. Participants will be randomly allocated in a 1:1 ratio to either the mVTEA management group or the routine management group. The mVTEA management group (n=128), also referred to as the experimental group, will receive patient-centered VTE management facilitated by the mVTEA app after discharge. In contrast, the routine management group (n=128), also known as the control group, will be administered with conventional postdischarge management according to local clinical practice. All of the above management will be performed for 3 months following discharge. Our study is scheduled to commence in August 2024 and conclude in November 2025. Recruitment began in August 2024 and is anticipated to conclude by June 2025. Data analysis is planned to commence in October 2025 after the follow-up data collection. The analysis is expected to be completed by November 2025. The protocol conforms to the SPIRIT (Standard Protocol Items: Recommendations for Interventional Trials) 2013 statement [37]. The latest protocol (version 1.0) was dated March 1, 2024. The trial was registered on Clinicaltrials.gov (NCT06350331).

### Participants and Recruitment

Potentially suitable candidates for the study will initially be screened from the inpatients in departments at high risk of VTE through the clinical decision support system for VTE risk assessment and integrated care in our hospital [38]. Based on the initial screening list, a final identification of whether the patient meets the study recruitment needs will be made on the day the patient is discharged from the hospital. Patients who fulfill the recruitment criteria and are willing to participate will receive comprehensive information about the entire study protocol, including its benefits and potential risks. Once patients agree to participate, they will be directed to a web-based informed consent document or, depending on the subject’s wishes, a paper version of the informed consent document will be given at the same time. This recruitment process will be carried out with the utmost respect for the individual preferences of each participant. In addition, we assure that the rights of participants to withdraw from the study will be upheld throughout all phases of the clinical trial. The inclusion and exclusion criteria of the study are shown in Textbox 1.

**Inclusion and exclusion criteria.**

**Inclusion criteria**

- Inpatients ≥18 years of age at admission.
- Previous or current definitive diagnosis of deep vein thrombosis (DVT), pulmonary embolism (PE), both by imaging, or high risk of venous thromboembolism (VTE) at discharge: Padua score ≥4 for medical patients and Caprini score ≥5 for surgical patients.
- Signed informed consent.

**Exclusion criteria**

- Mental disorder or a combination of other serious diseases leading to incapacity for independent living.
- Inability to use smartphones, computer tablets, and other smart devices.
- Being pregnant or breastfeeding.
- Have participated in similar trials or are undergoing other clinical trials.

### Randomization

Stratified block randomization was used to allocate participants. Participants were divided into 4 strata based on 2 factors: age (≥60 years and <60 years) and their VTE diagnostic status at the time of recruitment. The strata consist of (1) patients aged ≥60 years diagnosed with VTE, (2) patients aged ≥60 years at high risk for VTE but not diagnosed, (3) patients aged <60 years diagnosed with VTE, and (4) patients aged <60 years at high risk for VTE but not diagnosed. Within each stratum, participants were assigned in a 1:1 ratio to either the mVTEA management group or the routine management group. This allocation was performed using a fixed block randomization method with a block size of 4. The random sequence was generated by a free, robust randomization app with a random seed number of 20240215 [39]. A web-based random allocation system was used to assign random numbers to each participant.

### Blinding

Due to the limitations of the protocol and the specific nature of mHealth management, it is not feasible to blind participants, their primary care physicians, and clinic physicians. However, to enhance the quality and reliability of this study, the researchers will be blinded during the efficacy assessment, data collection, data management, and statistical analysis of the results. Therefore, those responsible for efficacy assessment, data collection and management, and analysis of results will not be informed of trial group assignments and will not be permitted to communicate with each other or with participants about the treatment. Furthermore, to minimize potential bias introduced by unblinded participants or researchers, the study will include only individuals who have no conflict of interest.

### Intervention

#### Overview

The mVTEA management group will receive patient-centered VTE management facilitated by the mVTEA app based on the combination of HBM and KAP frameworks after discharge, while the routine management group will undergo conventional postdischarge management according to local clinical practice. In addition, VTE management using the mVTEA app is administered by certified thrombosis specialists and nurses with over 5 years of clinical experience and proficiency in operating the mVTEA app. Furthermore, regular training sessions and assessments on VTE prevention and treatment for health care professionals are conducted in our hospital, along with routine quality control meetings focused on VTE prevention and treatment. These measures ensure that all health care professionals in our hospital possess the necessary competencies to effectively implement VTE prevention and treatment.

#### Integrated VTE Management Pathway Within mVTEA

The long-term management of VTE is multifaceted, requiring individualized patient assessment and a nuanced approach to therapy. In this study, we have proposed the ABCDEF pathway for integrated management of VTE to complement mVTEA-assisted patient-centered management, thus minimizing the risk of VTE and its complications and ensuring patient safety and quality of life (QoL): (1) appropriate antithrombotic management; (2) bleeding risk management; (3) complication monitoring; (4) digital health management; (5) exercise and rehabilitation; and (6) facilitate the management of risk factors and comorbidities. Details of the integrated VTE management pathway are presented in Table 1.

**Table 1:** The ABCDEF pathway for integrated management of venous thromboembolism.

Pathway and key point			Process
**Appropriate antithrombotic management [13,40-46]**			
	Thrombosis risk management	Monitor patients’ thrombotic risk after discharge to facilitate thrombosis prevention, improve treatment decisions, and enhance patient self-management.	
	Anticoagulant therapy selection	Choose the appropriate anticoagulant (eg, warfarin or DOACs^a^) based on individual patient risk factors, renal function, and potential drug interactions.	
	Dose adjustment and monitoring	Conduct regular blood tests (eg, INR^b^ for warfarin and renal function tests for DOACs) and patient consultations to monitor therapeutic effects. Schedule follow-ups to reassess and adjust dosages based on test results and patient feedback.	
	Thromboprophylaxis	Assess patient risk factors to determine the need for prophylaxis, administer appropriate prophylactic measures, and monitor their effectiveness.	
**Bleeding risk management [13,14,40-47]**			
	Bleeding risk assessment	Conduct a thorough bleeding risk assessment using validated tools (eg, HAS-BLED^c^ score) before initiating anticoagulant therapy and reassess periodically.	
	Personalized anticoagulant therapy	Tailor anticoagulant therapy to the individual’s bleeding risk, opting for agents with lower bleeding risk profiles if necessary.	
	Bleeding precautions	Implement preventive measures such as avoiding unnecessary invasive procedures and managing concurrent medications that may increase bleeding risk.	
**Complication monitoring [13,44,48,49]**			
	Regular complication surveillance	Schedule regular check-ups and use diagnostic imaging (eg, Doppler ultrasound, CTPA^d^) to monitor for complications such as recurrent VTE^e^, PTS^f^, PPES^g^, or CTEPH^h^.	
	Patient self-monitoring	Encourage patients to report new or worsening symptoms promptly, and provide them with clear instructions on what to look out for.	
**Digital health management [22-24,50]**			
	Telemedicine	Implement telemedicine platforms for regular follow-ups, reducing the need for in-person visits and enabling remote monitoring of anticoagulation therapy.	
	mHealth^i^ apps	Use mHealth apps that allow patients to track their medication adherence, report symptoms, and receive reminders for follow-up appointments.	
	Wearable devices	Encourage the use of wearable devices that monitor vital signs (eg, heart rate and oxygen saturation) to detect early signs of complications.	
**Exercise and rehabilitation [51,52]**			
	Personalized exercise programs	Develop individualized exercise programs that take into account the patient’s overall health status, VTE history, and comorbid conditions.	
	Supervised rehabilitation	Initially, provide supervised rehabilitation to ensure safe exercise practice and adjust the intensity and type of exercise as needed.	
	Long-term physical activity	Encourage long-term adherence to physical activity guidelines to improve health outcomes.	
**Management of vascular risks and comorbidities [53-57]**			
	Comprehensive vascular risk assessment	Conduct a thorough assessment to identify and manage modifiable vascular risk factors such as obesity, smoking, hypertension, and diabetes. Monitor for cardiac arrhythmias such as atrial fibrillation and obstructive sleep apnea.	
	Lifestyle modifications	Provide education and support for lifestyle changes, including dietary modifications, smoking cessation programs, and weight management strategies.	
	Integrated care for cardiovascular risks and comorbidities	Coordinate care among specialists to manage comorbidities effectively, ensuring that treatments for VTE are compatible with other medical conditions and medications.	

^a^DOACs: direct oral anticoagulants.

^b^INR: international normalized ratio.

^c^HAS-BLED: hypertension, abnormal renal or liver function, stroke, bleeding history or predisposition, labile INR, elderly (age ≥ 65 years), drugs or alcohol concomitantly.

^d^CTPA: computed tomography pulmonary angiography.

^e^VTE: venous thromboembolism.

^f^PTS: postthrombotic syndrome.

^g^PPES: postpulmonary embolism syndrome.

^h^CTEPH: chronic thromboembolic pulmonary hypertension.

^i^mHealth: mobile health.

#### The HBM and KAP-Combining Framework

**Figure 1:**
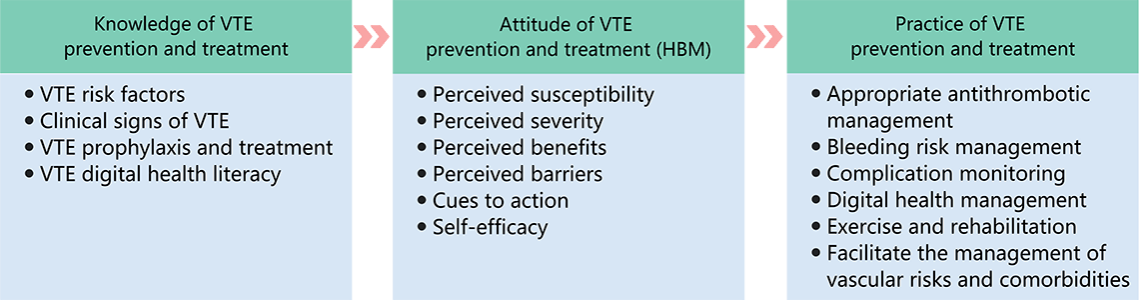
Combined framework based on the health belief model and the knowledge, attitude, and practice approach. HBM: health belief model; VTE: venous thromboembolism.

The HBM suggests that people’s perceptions of the severity and susceptibility to a health issue, along with the perceived benefits and barriers to taking preventive action, influence their decisions to adopt health behaviors. The KAP highlights the sequential relationship among knowledge, attitudes, beliefs, practices, or behaviors. It posits that increased knowledge leads to changes in attitudes, which influence practices or behaviors. The key components of the HBM and the KAP are shown in Textbox 2. This study further extends the framework of the KAP by incorporating the HBM within the attitude domain. Through an in-depth analysis of the factors influencing the adoption of health behaviors, we believe that it will improve patients’ health beliefs, increase self-efficacy in health behaviors, and promote patient-centered management. Furthermore, this study has taken an additional step by incorporating the ABCDEF pathway for integrated management of VTE into the practice domain of the KAP, thereby assessing patient health behaviors more comprehensively. Figure 1 shows the combined HBM and KAP framework.

**The key components of the health belief model and the knowledge-attitude-practice frameworks.**

**Health belief model**

- Perceived susceptibility: belief about the likelihood of getting a disease or condition.
- Perceived severity: belief about the seriousness of the disease or condition.
- Perceived benefits: belief in the effectiveness of taking action to reduce the risk or severity.
- Perceived barriers: beliefs about the obstacles to taking the recommended action.
- Cues to action: triggers that prompt individuals to take action, such as symptoms or media campaigns.
- Self-efficacy: confidence in one’s ability to take the recommended action.

**Knowledge-attitude-practice**

- Knowledge: awareness or understanding of health-related information and facts.
- Attitude: beliefs, feelings, and values toward a particular health issue or behavior.
- Practice: the actual behaviors or actions taken in response to health knowledge and attitudes.

#### mVTEA-Facilitated Patient-Centered VTE Management

**Figure 2:**
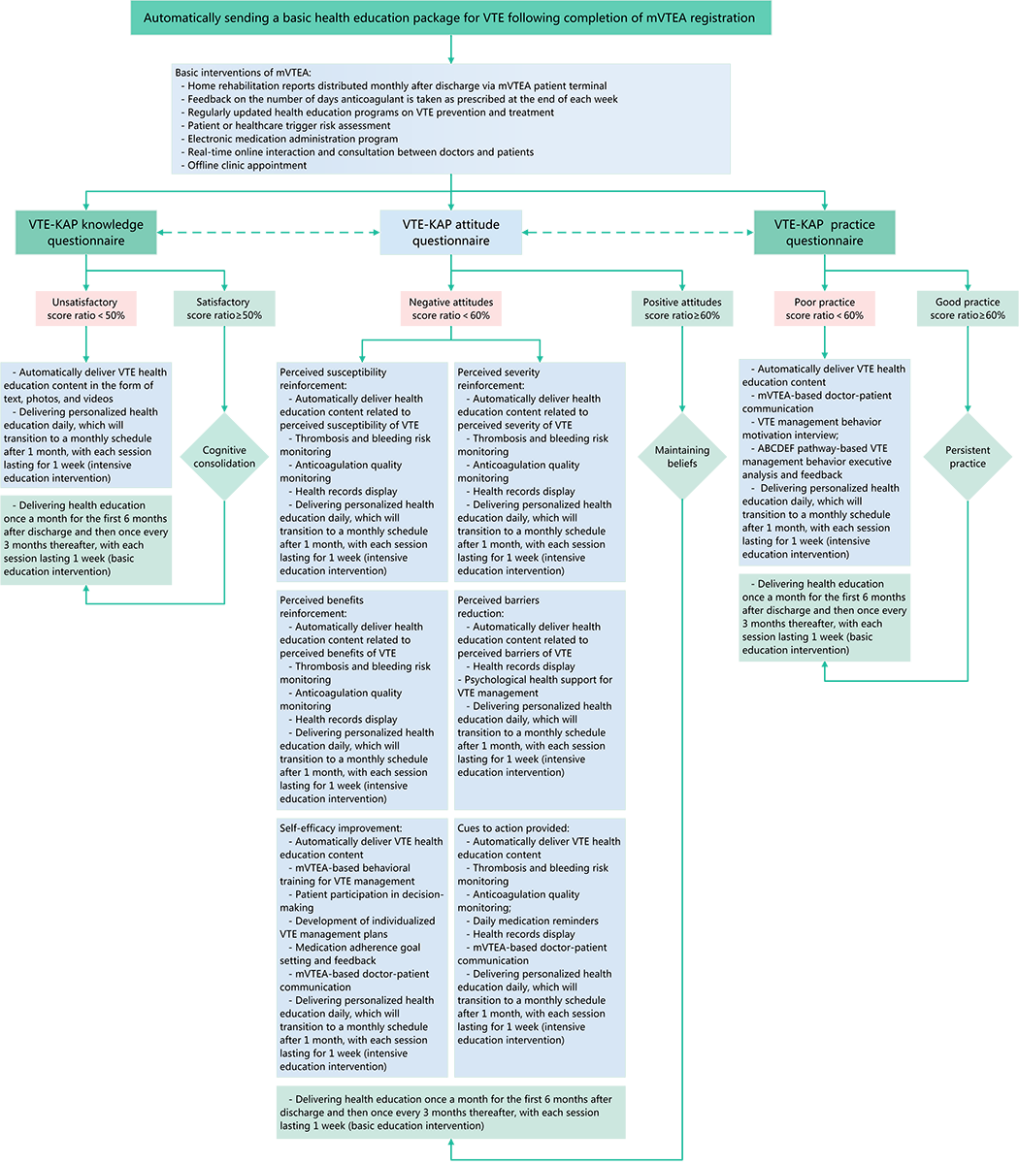
Specific intervention process of the mHealth app for patient-centered venous thromboembolism management. KAP: knowledge, attitude, and practice; mVTEA: smart technique-assisted patient-centered care mHealth app for managing venous thromboembolism; VTE: venous thromboembolism.

To achieve the goal of mVTEA-facilitated patient-centered VTE management, an mVTEA-assisted patient health education program has been structured around the joint HBM and KAP framework as shown in Figure 2. To begin with, in a pilot study, we have developed a structured VTE-KAP questionnaire (Multimedia Appendix 1), guided by this joint theory framework, via 2 rounds of Delphi surveys among the National VTE Prevention and Treatment Program Office Expert Group and the VTE Prevention and Treatment Nursing Expert Group of the Chinese People’s Liberation Army General Hospital. This questionnaire demonstrated an overall Cronbach α coefficient of 0.959, with domain-specific coefficients of 0.885 for knowledge, 0.942 for attitudes, and 0.970 for practices. In addition, the overall split-half reliability coefficient was 0.834, with domain-specific coefficients of 0.879 for knowledge, 0.770 for attitudes, and 0.863 for practices.

The VTE-KAP questionnaire is a self-administered questionnaire that includes demographic characteristics and KAP of patients toward VTE prevention and treatment. It is intended to evaluate the efficacy of health education programs and to inform the deployment of mVTEA-based health education strategies. This questionnaire consists of a total of 53 questions with an overall score range of 41-283. The knowledge domain contains seven questions to assess patients’ levels of knowledge regarding VTE prevention and treatment. The total score of knowledge for each study participant ranged from 7 to 53. The attitude domain contains 20 questions to assess patients’ attitudes toward VTE prevention and treatment. Each question was scored, and the ﬁnal attitude score ranged between 20 and 100. The practice domain contained 26 questions with a score range of 21-130 for assessing the implementation of VTE prevention and treatment behaviors in the study patients. The higher the score, the higher the level of knowledge, attitude, and practice.

To grade the KAP about VTE prophylaxis and treatment, the VTE-KAP questionnaire score is calculated according to the score ratio: score ratio=item score/total score of the item×100%. According to the previous study [58], a score ratio of ≥50% in the knowledge domain is classified as satisfactory and <50% as unsatisfactory; a score ratio of ≥60% in the attitude domain is classified as positive attitudes and <60% as negative attitudes; and a score ratio of ≥60% in the practice domain is classified as a good level of practice and <60% is classified as poor practice.

**Figure 3:**
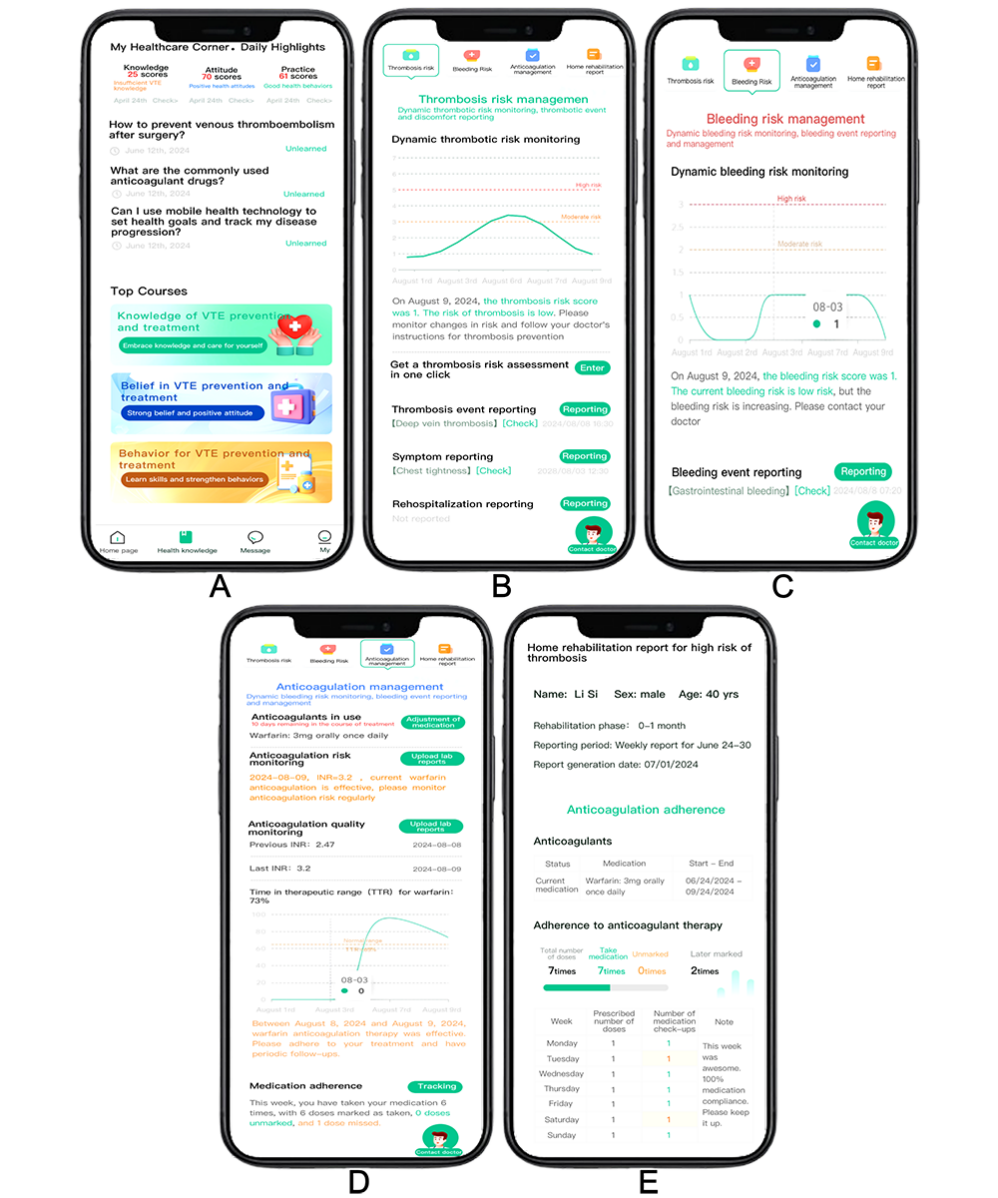
Core functional interface of the mHealth app for patient-centered venous thromboembolism management. (A) Health education program, (B) thrombosis risk assessment, (C) bleeding risk assessment, (D) anticoagulation management, and (E) home rehabilitation report.

These VTE-KAP assessment outcomes determine the subsequent interventions delivered via the mVTEA app. Initially, upon registration, the mVTEA app delivers a standardized VTE education package containing 3 videos and 4 graphic resources covering VTE fundamentals, risk factors, symptoms, and prevention and treatment strategies. Following this initial phase, interventions are personalized based on the individual’s VTE-KAP scores. Tailored components include specific education schedules (basic or intensive), medication reminders, adherence feedback, risk monitoring features, access to electronic health records, and specialist communication channels. In addition, the mVTEA patient terminal automatically dispatches all health education questionnaires and home-rehabilitation reports according to the schedule specified in Table 2. The detailed workflow of this mVTEA-facilitated patient-centered VTE management strategy is presented in Figure 2, and the core functional interface of the mVTEA app is shown in Figure 3.

**Table 2:** Distribution schedule of home rehabilitation reports and health education questionnaires for mHealth app-assisted, patient-centered venous thromboembolism management.

Timepoint	At discharge	1st month	2nd month	3rd month	6th month	9th month	12th month	>1 year
Home rehabilitation reports	None	✓	✓	✓	Once a month	Once a month	Once a month	Once a month
Knowledge questionnaire	✓	None	None	✓	✓	None	✓	Once every 6 months
Attitude questionnaire	✓	✓	✓	✓	✓	✓	✓	Once every 6 months
Practice questionnaire	✓	None	None	✓	✓	None	✓	Once every 6 months

#### Routine VTE Management

The routine VTE management group will receive only conventional postdischarge management according to local clinical practice, including basic health advice appropriate for the prevention and treatment of VTE. The textual and graphical materials comprising the aforementioned basic package for VTE-related health education in the mVTEA app will be printed and distributed to patients within the routine management group on the day of discharge for home-based study. The video courses in the health education basic package can be accessed by scanning the QR code.

### Outcome Measures

#### Primary Outcome

The primary outcome of this study is the difference in patients’ KAP on VTE at 3-month follow-up between the mVTEA management group and the routine management group.

#### Secondary Outcomes

Secondary outcomes include scores on each domain of the VTE-KAP questionnaire (knowledge domain, attitude domain, practice domain), QoL, VTE events, chronic thromboembolic pulmonary hypertension, chronic thromboembolic pulmonary disease, post-PE syndrome [13,48], International Society on Thrombosis and Hemostasis (ISTH) defined major bleeding events [59], VTE-related hospitalizations, VTE-related rehospitalizations, all-cause deaths, PE-related deaths, and new-onset of atrial fibrillation (AFI) or atrial flutter (AFL) at 3-month follow-up. For patients with more than 1 event, only the first event will be counted. A total of 3 investigators who are unaware of patients’ group assignments will adjudicate all endpoints.

Generic, nondisease-speciﬁc health-related QoL is assessed using the EQ-5D-5L questionnaire and its corresponding visual analog scale [60]. Brieﬂy, the EQ-5D-5L generates an overall index that ranges from 0 (lowest generic QoL) to 1 (highest generic QoL) and is calculated based on country-speciﬁc reference value sets. The EQ-5D-5L health index was calculated with the value set for China. The EuroQol visual analog scale ranges from 0 to 100, with higher scores indicating better health.

VTE events are categorized into 3 groups: new-onset VTE, hospital-acquired VTE (HA-VTE), and recurrent VTE. VTE that occurred for the first time during the study period is classified as new-onset VTE. HA-VTE is defined as any new-onset VTE that has occurred within 90 days of hospital discharge [4]. Recurrent VTE is defined as the appearance of new evidence of VTE after acute VTE has been treated in the acute phase (2 weeks) with significant clinical improvement in signs and symptoms. According to the time of VTE recurrence, it is further categorized into early VTE recurrence (within 3 months after the last VTE occurrence) and late VTE recurrence (more than 3 months after the last VTE occurrence) [61]. Hospitalization due to the new-onset VTE will be documented as VTE-related hospitalizations, while rehospitalization due to VTE recurrence, progression, or complications arising from VTE treatment will be classified as VTE-related rehospitalizations. All-cause death refers to any death occurring during the study period, regardless of the cause. PE-related death is defined as death explicitly caused by PE.

New-onset AFI or AFL is defined as an episode of AFI or AFL lasting at least 30 seconds, first detected by devices such as smart wearable devices or ambulatory electrocardiograms, or initially diagnosed during a medical visit and documented in outpatient charts or inpatient hospital records.

### Data Collection and Management

Table 3 presents the detailed schedule for data collection and assessments. Baseline demographic and clinical data will be extracted from the hospital information system. Additional data—including mHealth use, electronic health literacy, EQ-5D-5L, and VTE-KAP—will be collected at discharge and follow-up via web-based questionnaires for the routine group and through the mVTEA platform for the intervention group. The Chinese version of the electronic health literacy scale (C-eHEALS) will be used to evaluate participants’ eHealth literacy [62]. Follow-up at 3 months postdischarge will be conducted through outpatient visits, telephone interviews, or web-based interviews via the mVTEA platform (for the intervention group). All collected data will be deidentified and recorded in electronic case report forms. Data will be securely stored on password-protected servers with restricted access, and sharing with third parties will require authorization from the corresponding author. The quality control team will routinely verify data accuracy and reliability; upon confirmation, the data will be locked to prevent further modifications. The finalized database will be provided to statistical analysts as specified in the statistical analysis plan. All data collectors and managers will undergo standardized training and assessment before participating in the study.

**Table 3:** Study protocol, schedule, and timing of data collection.

Activity or assessment	Screening (during hospitalization, t_1_)	Enrollment (at discharge, 0)	Study visit (at discharge [intervention], t_1_)	Follow-up (3rd month, t_2_)
Prescreening consent	✓			
Eligibility screen	✓			
Informed consent		✓		
Randomization		✓		
Demographic information		✓		
Medical history		✓		
mHealth use		✓		
C-eHEALS^a^		✓		
VTE^b^-KAP^c^ questionnaire		✓		✓
EQ-5D-5L		✓		✓
Interventions				
Basic health education			✓	
mVTEA^d^-assisted management			✓	✓
Routine management			✓	✓
VTE event				✓
CTEPH^e^				✓
CTEPD^f^				✓
PPES^g^				✓
Bleeding event				✓
VTE-related hospitalization				✓
VTE-related rehospitalization				✓
Death				✓
New-onset of AFI^h^ or AFL^i^				✓
Adverse event				✓

^a^C-eHEALS: Chinese version of the electronic health literacy scale.

^b^VTE: venous thromboembolism.

^c^KAP: knowledge, attitude, and practice.

^d^mVTEA: smart technique-assisted patient-centered care mHealth app for managing venous thromboembolism.

^e^CTEPH: chronic thromboembolic pulmonary hypertension.

^f^CTEPD: chronic thromboembolic pulmonary disease.

^g^PPES: postpulmonary embolism syndrome.

^h^AFI: atrial fibrillation.

^i^AFL: atrial flutter.

### Quality Control

To ensure the quality of the study, all researchers will undergo uniform and standardized training before the study’s commencement. Researchers will be assessed, and only those who meet the qualifications will be allowed to participate. Structured follow-ups will be conducted by staff from the specialized follow-up office. In addition, the collected data will be regularly monitored monthly by the research supervisor to ensure the authenticity and reliability of the study results.

### Statistics

#### Sample Size and Power Considerations

This study used a stratified block randomization method with 4 strata. Literature suggests an average of 50-100 patients per stratum for stratified randomization [63]. Previous studies in China have reported that the coefficient of variation for VTE-KAP questionnaire scores among patients at risk of VTE ranges from 0.1 to 0.12. In this study, a coefficient of variation of 0.1 was assumed, enrolling 50 patients per stratum, resulting in a total of 200 patients (100 per group). At a 2-sided significance level of .05, this design yields 94% statistical power to detect a 5% difference in VTE-KAP questionnaire scores between the two groups. Even with a 2-sided significance level of 0.025, the setup maintains 90% statistical power to detect the same 5% difference. Considering a 20% nonresponse rate, the final sample size was adjusted to 64 patients per stratum, leading to a total of 256 patients (128 in each group). The sample size was calculated by using Power Analysis and Sample Size software (version 19.4.1; NCSS, LLC).

#### Statistical Analyses

In this study, we define 2 analysis populations: full analysis set (FAS) and intention-to-treat (ITT). FAS is the set of all patients who have received VTE prevention and treatment intervention at least once after randomization. The ITT set will include all randomly assigned patients, regardless of whether they completed the study or followed the study protocol. FAS will be the primary analysis population, and ITT will provide supportive results. Missing data will be handled using the multiple imputation method. Since the study outcomes already include assessments of safety data, the safety analysis set is not performed. Sensitivity analysis will be conducted by comparing the results of the ITT and FAS analyses.

Continuous variables will be presented as mean and SD, or as median and IQR for variables not following a normal distribution. Categorical variables will be presented as counts and percentages. Differences in baseline demographic and clinical characteristics, as well as survey data between groups, will be assessed using the Student *t* test or the Mann-Whitney *U* test for continuous variables, depending on distributional assumptions. Categorical variables will be analyzed using the chi-square test or Fisher exact test, as applicable.

To account for the repeated measurements of EQ-5D-5L and KAP survey data collected at discharge and the 3-month follow-up, we will use a linear mixed-effects model for repeated measures. The model will include fixed effects for the treatment group (mVTEA management vs routine management), time point (discharge vs 3-month follow-up), the interaction between treatment group and time point, the baseline score of the respective survey (EQ-5D-5L or KAP), and the prestratification factors (VTE diagnosis status at discharge: Yes or No; age: ≥60 years vs <60 years). Patients and their hospitalization units will be included as random effects. The key parameter of interest for evaluating the intervention effect over time will be the group-by-time interaction term. The Cox proportional hazards model will be used to compare the hazard of the event (time to event) between groups during the follow-up period, taking into account the prestratification factors. The hazard ratio and its corresponding 95% CI will be calculated using this model. Statistical significance is set at *P*<.05 (2-sided). SPSS (version 22.0; IBM Corp) will be used for statistical analysis.

### Ethical Considerations

This study will be conducted by the principles outlined in the Declaration of Helsinki, ensuring that no additional harm or risks are imposed upon the participants. The ethics committee of the Sixth Medical Center of the Chinese People’s Liberation Army General Hospital has already provided ethics approval (HZKY-PJ-2024-8). Before the commencement of the clinical trial, all participants will receive comprehensive information about the trial’s purpose, the procedures to be undertaken, the anticipated duration, and the potential risks and benefits. They will then voluntarily provide written informed consent. To maintain participant confidentiality, real names will not be used in case reports, and participants will retain the right to withdraw from any aspect of the study at any time. Research findings will be disseminated through publication in peer-reviewed journals and presentations at provincial, national, and international conferences or forums.

## Results

**Figure 4:**
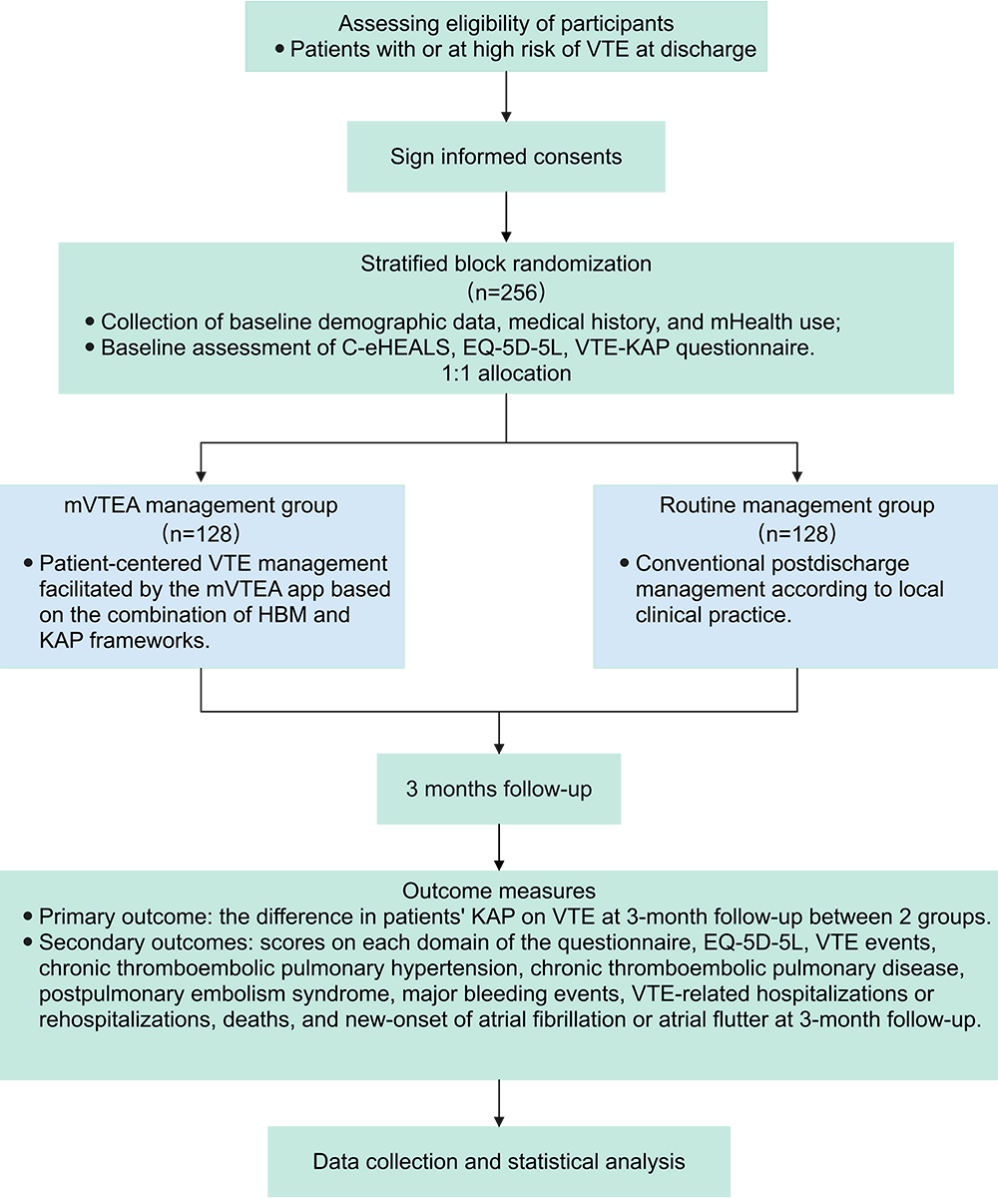
Flowchart of the trial. C-eHEALS: Chinese version of electronic health literacy scale; HBM: health belief model; KAP: knowledge, attitude, and practice; mHealth: mobile health; mVTEA: smart technique-assisted patient-centered care mHealth app for managing venous thromboembolism; VTE: venous thromboembolism.

Figure 4 describes the study flowchart. Participants are currently being recruited. The first participant was enrolled in August 2024, marking the official start of the study. The recruitment process is expected to be completed in June 2025. As of the submission of the paper, 185 patients were enrolled in this clinical trial. All included patients were followed up according to the outlined schedule.

## Discussion

### Expected Outcomes

The SmaVTE study is an innovative study designed to address the multifaceted challenges of VTE management through the integration of smart technology. This trial aims to evaluate the effectiveness of the mVTEA app in VTE management, which is an mHealth app designed to enhance patient-centered care through the joint use of HBM and KAP theoretical frameworks. This study hypothesizes that such theory-based mHealth interventions can improve the KAP level for patients with or at risk of VTE while ensuring their safety compared to traditional face-to-face VTE management.

Unlike previous mHealth studies that focused mainly on patients with warfarin therapy, targeting patients with AFI and heart valve disease [26,64-66], the SmaVTE study expands the target population to include both patients at risk for VTE and those already diagnosed, as well as patients using direct oral anticoagulants (DOACs). This comprehensive scope is designed to reflect real-world clinical practice and address emerging challenges associated with DOAC management, such as renal impairment, drug interactions, and the absence of specific reversal agents [45,67]. Integrating the ABCDEF pathway into the KAP framework further supports a comprehensive and dynamic approach to VTE care, encompassing not only anticoagulation management but modifiable risk factors, long-term complications, and comorbidities. If proven effective, findings from this trial may have significant implications for clinical practice, supporting more proactive and personalized approaches to VTE prevention and treatment.

The theoretical foundation of the mVTEA app is grounded in the principles of patient-centered care and behavioral science theories. Patient-centered care emphasizes tailoring medical care to individual patient needs, preferences, and values [15,16]. Behavioral science theories, such as the HBM and the KAP theories, suggest that empowering patients with information and involving them in their care can significantly enhance motivation and adherence to treatment protocols [31-34]. The mechanisms by which the mVTEA app may influence behavior change can be attributed to several factors, including goal setting, real-time feedback, personalized reminders, and tailored health education programs. These features are designed to facilitate continuous engagement and allow patients to monitor their progress, thereby enhancing motivation and adherence.

### Limitations

There were some limitations to this study. First, the applicability of the results may be confined to the population studied and may not fully extrapolate to other patient groups, such as those with AFI or heart valve disease. Second, the study encompasses only those patients who can use smartphones or computer tablets, which introduces a potential bias related to the participants’ digital literacy. To mitigate this effect, standardized training on the application’s use will be provided following the patient’s registration on the mVTEA app. Third, the experimental protocol could not implement double blinding, a limitation inherent to the specificity of mHealth interventions. To ensure the reliability of the data, blind statistical analysis will be conducted, and roles among interventionists, efficacy evaluators, and statisticians will be distinctly segregated. Finally, the clinical outcomes, designated as secondary endpoints in this study, require validation through subsequent research.

### Conclusion

The SmaVTE study may contribute to advances in VTE prevention and treatment. By integrating smart technology with patient-centered care and applying well-established theoretical frameworks, the study aims to enhance the KAP of patients at risk for or diagnosed with VTE while improving clinical outcomes. In addition, this study provides a novel approach for the long-term management of VTE. The results from this trial could have significant implications for clinical practice and may encourage further research into the integration of smart technologies with theory-based behavior change techniques.

## Appendix

Multimedia Appendix 1 Additional material.

## Abbreviations

AFI: atrial fibrillation
AFL: atrial flutter
C-eHEALS: Chinese version of the electronic health literacy scale
DOAC: direct oral anticoagulant
FAS: full analysis set
HBM: health belief model
ISTH: International Society on Thrombosis and Hemostasis
ITT: intention-to-treat
KAP: knowledge, attitudes, and practices
mHealth: mobile health
mVTEA: smart technique–assisted patient-centered care mHealth app for managing venous thromboembolism
PE: pulmonary embolism
QoL: quality of life
SmaVTE: smart technology facilitated patient-centered venous thromboembolism management
SPIRIT: Standard Protocol Items: Recommendations for Interventional Trials
VTE: venous thromboembolism

## Data Availability

The datasets generated and analyzed in this study are available upon request from the corresponding author. At present, data sharing is not applicable.
