# Supplementary material for: Smart Technology Facilitated Patient-Centered Venous Thromboembolism Management (the SmaVTE Study): Protocol for a Randomized Controlled Trial
Source: JMIR Res Protoc. 2025 Jun 5;14:e67254. doi: 10.2196/67254 (PMC12179571; doi:10.2196/67254)
Supplement: Multimedia Appendix 1 [file resprot_v14i1e67254_app1.docx]

**Supplementary Materials**

**Appendix A:** Questionnaire on patients' knowledge, attitudes and practices regarding the prevention and treatment of venous thromboembolism

**Appendix B:** All items from the World Health Organisation Trial dataset

**Appendix C:** Informed Consent Form

**Appendix D:** Reporting checklist for protocol of a clinical trial based on the SPIRIT guidelines

**Appendix A:** Questionnaire on patients' knowledge, attitudes and practices regarding the prevention and treatment of venous thromboembolism

**第一部分 《患者静脉血栓栓塞症防治-知识问卷》**

请您根据个人经验和知识在相应的框中进行勾选

K1.1您认为患有静脉血栓栓塞症可能会带来以下哪些危害？ [多选题]

| □K1.1.1.影响身体康复 |
| --- |
| □K1.1.2.延长住院时间 |
| □K1.1.3.增加医疗费用 |
| □K1.1.4.合并发生与静脉血栓栓塞症相关的其他疾病 |
| □K1.1.5.降低生活质量 |
| □K1.1.6.带来生命危险 |

K2.1您认为下列哪些因素可能会增加发生静脉血栓栓塞症的风险？ [多选题]

| □K2.1.1.高龄 |
| --- |
| □K2.1.2.肥胖 |
| □K2.1.3.长时间乘坐交通工具 |
| □K2.1.4.长时间卧床、久坐或行动不便 |
| □K2.1.5.近期创伤或外科手术史 |
| □K2.1.6.下肢骨折 |
| □K2.1.7.口服避孕药或激素药物 |
| □K2.1.8.怀孕和产后 |
| □K2.1.9.合并患有多种疾病（包括高血压、高血脂、冠心病、脑卒中、糖尿病、心肺功能不全等） |
| □K2.1.10.急性感染或风湿性疾病 |
| □K2.1.11.肿瘤 |
| □K2.1.12.以前患过静脉血栓栓塞症 |

K3.1您认为下肢深静脉血栓形成可能有以下哪些临床表现？ [多选题]

| □K3.1.1.腿部肿胀 |
| --- |
| □K3.1.2.腿部疼痛 |
| □K3.1.3.腿部发热 |
| □K3.1.4.腿部发凉 |
| □K3.1.5.腿部皮肤颜色改变 |
| □K3.1.6.浅静脉曲张 |

K3.2您认为肺栓塞可能有以下哪些临床表现？ [多选题]

| □K3.2.1.气短 |
| --- |
| □K3.2.2.剧烈咳嗽 |
| □K3.2.3.胸闷 |
| □K3.2.4.胸痛 |
| □K3.2.5.咳血 |
| □K3.2.6.晕厥 |
| □K3.2.7.心慌 |

K4.1您认为采取以下哪些措施可以预防静脉血栓栓塞症的发生？ [多选题]

| □K4.1.1.多喝水（在身体情况允许的情况下） |
| --- |
| □K4.1.2.戒烟戒酒 |
| □K4.1.3.保持良好饮食习惯，食用纤维含量高、低胆固醇且低脂的各类健康食物 |
| □K4.1.4.卧床期间多做肢体活动，如踝关节活动、下肢关节活动等 |
| □K4.1.5.卧床期间抬高下肢，按摩肢体，必要时使用康复器材辅助运动 |
| □K4.1.6.尽早下床活动（在身体情况允许的情况下） |
| □K4.1.7.平时规律锻炼（在身体情况允许的情况下） |
| □K4.1.8.使用逐级加压袜（弹力袜） |
| □K4.1.9.使用间歇充气加压装置（足底压力泵） |
| □K4.1.10.使用华法林、利伐沙班、艾多沙班、达比加群、低分子肝素、普通肝素等抗凝药物 |

K4.2您认为下列哪些是静脉血栓栓塞症的治疗措施？ [多选题]

| □K4.2.1.使用华法林、利伐沙班、艾多沙班、达比加群、低分子肝素、普通肝素等抗凝药物 |
| --- |
| □K4.2.2.使用尿激酶、链激酶、阿替普酶等溶解血栓药物 |
| □K4.2.3.下腔静脉滤器植入、血栓抽吸、经导管碎栓/取栓/溶栓等介入治疗措施 |
| □K4.2.4.高血压、高血脂、冠心病、脑卒中、糖尿病等伴随疾病的管理 |
| □K4.2.5.康复锻炼 |

K5.1下列这些数字健康管理技术您听说过吗？ [多选题]

| □K5.1.1.远程医疗会诊 |
| --- |
| □K5.1.2.手机或平板电脑软件辅助健康管理 |
| □K5.1.3.佩戴智能设备监测健康（例如智能手环、智能手表、活动跟踪器） |
| □K5.1.4.医患交流网络平台 |
| □K5.1.5.医学智能对话助手提供医疗咨询 |
| □K5.1.6.计算机模拟人类思维辅助疾病诊断和治疗 |
| □K5.1.7.数字化存储个人健康档案（例如电子病历） |

**Part 1: Questionnaire on Patients’ Knowledge Regarding the Prevention and Treatment of Venous Thromboembolism**

Please select the appropriate boxes based on your personal experience and knowledge.

**K1.1 What do you think are the potential harms of venous thromboembolism? [Multiple choice question]**

□ K1.1.1. Impairment of physical recovery

□ K1.1.2. Prolonged hospital stay

□ K1.1.3. Increased medical costs

□ K1.1.4. Concomitant occurrence of other diseases related to venous thromboembolism

□ K1.1.5. Decreased quality of life

□ K1.1.6. Life-threatening risk

**K2.1 Which of the following factors do you think might increase the risk of venous thromboembolism? [Multiple choice question]**

□ K2.1.1. Advanced age

□ K2.1.2. Obesity

□ K2.1.3. Prolonged travel by vehicle

□ K2.1.4. Prolonged bed rest, sitting, or immobility

□ K2.1.5. Recent trauma or surgical history

□ K2.1.6. Lower limb fracture

□ K2.1.7. Oral contraceptives or hormone medications

□ K2.1.8. Pregnancy and postpartum

□ K2.1.9. Having multiple coexisting conditions (including hypertension, hyperlipidemia, coronary heart disease, stroke, diabetes, cardiopulmonary insufficiency, etc.)

□ K2.1.10. Acute infection or rheumatic diseases

□ K2.1.11. Tumor

□ K2.1.12. Previous history of venous thromboembolism

**K3.1 What do you think are the possible clinical manifestations of deep vein thrombosis in the lower limbs? [Multiple choice question]**

□ K3.1.1. Leg swelling

□ K3.1.2. Leg pain

□ K3.1.3. Leg warmth

□ K3.1.4. Leg coolness

□ K3.1.5. Changes in skin color of the leg

□ K3.1.6. Superficial varicose veins

**K3.2 What do you think are the possible clinical manifestations of pulmonary embolism? [Multiple choice question]**

□ K3.2.1. Shortness of breath

□ K3.2.2. Severe coughing

□ K3.2.3. Chest tightness

□ K3.2.4. Chest pain

□ K3.2.5. Hemoptysis (coughing up blood)

□ K3.2.6. Syncope

□ K3.2.7. Palpitations

**K4.1 What measures do you think can prevent venous thromboembolism? [Multiple choice question]**

□ K4.1.1. Drinking more water (as permitted by your condition)

□ K4.1.2. Quitting smoking and alcohol

□ K4.1.3. Maintaining good dietary habits, such as eating high-fiber, low-cholesterol, and low-fat healthy foods

□ K4.1.4. Performing limb exercises during bed rest, such as ankle and lower limb joint movements

□ K4.1.5. Elevating the lower limbs during bed rest, massaging the limbs, and using rehabilitation equipment if necessary

□ K4.1.6. Getting out of bed as soon as possible (if permitted by your condition)

□ K4.1.7. Regular exercise (if permitted by your condition)

□ K4.1.8. Using graduated compression stockings (compression stockings)

□ K4.1.9. Using intermittent pneumatic compression devices (foot pumps)

□ K4.1.10. Using anticoagulants such as warfarin, rivaroxaban, edoxaban, dabigatran, low molecular weight heparin, or standard heparin

**K4.2 What do you think are the treatment measures for venous thromboembolism? [Multiple choice question]**

□ K4.2.1. Using anticoagulants such as warfarin, rivaroxaban, edoxaban, dabigatran, low molecular weight heparin, or standard heparin

□ K4.2.2. Using thrombolytic drugs such as urokinase, streptokinase, or alteplase

□ K4.2.3. Interventional treatments such as inferior vena cava filter implantation, thrombectomy, catheter-directed thrombolysis/thrombectomy, etc.

□ K4.2.4. Management of comorbid conditions such as hypertension, hyperlipidemia, coronary heart disease, stroke, diabetes, etc.

□ K4.2.5. Rehabilitation exercises

**K5.1 Have you heard of the following digital health management technologies? [Multiple choice question]**

□ K5.1.1. Telemedicine consultation

□ K5.1.2. Health management applications on smartphones or tablets

□ K5.1.3. Health monitoring using wearable devices (e.g., smart bands, smartwatches, activity trackers)

□ K5.1.4. Patient-doctor communication platforms

□ K5.1.5. Medical intelligent conversation assistants providing medical advice

□ K5.1.6. Computer simulations aiding in disease diagnosis and treatment

□ K5.1.7. Digital storage of personal health records (e.g., electronic medical records)

**第二部分 《患者静脉血栓栓塞症防治-态度问卷》**

请选择最符合您当前想法的选项

A1 感知VTE的易感性

|  | 非常不同意 | 不同意 | 不确定 | 同意 | 非常同意 |
| --- | --- | --- | --- | --- | --- |
| A1.1 我认为定期评估血栓风险是必要的 | ○ | ○ | ○ | ○ | ○ |
| A1.2 我认为血栓风险是动态变化的 | ○ | ○ | ○ | ○ | ○ |
| A1.3 我认为若出现新的身体不适症状，或既往存在的身体不适症状加重时，应尽快就医 | ○ | ○ | ○ | ○ | ○ |

A2 感知VTE的严重性

|  | 非常不同意 | 不同意 | 不确定 | 同意 | 非常同意 |
| --- | --- | --- | --- | --- | --- |
| A2.1 我认为患有静脉血栓栓塞症会影响我的身体健康 | ○ | ○ | ○ | ○ | ○ |
| A2.2 我认为患有静脉血栓栓塞症会使我有心理压力 | ○ | ○ | ○ | ○ | ○ |
| A2.3我认为患有静脉血栓栓塞症会影响到我以后的正常工作和生活 | ○ | ○ | ○ | ○ | ○ |
| A2.4 我认为患有静脉血栓栓塞症会给我的家庭带来经济负担 | ○ | ○ | ○ | ○ | ○ |

A3 感知健康行为的益处

|  | 非常不同意 | 不同意 | 不确定 | 同意 | 非常同意 |
| --- | --- | --- | --- | --- | --- |
| A3.1进行静脉血栓栓塞症防治可以减少该疾病及其并发症的发生 | ○ | ○ | ○ | ○ | ○ |
| A3.2进行静脉血栓栓塞症防治可以增加我的安全感 | ○ | ○ | ○ | ○ | ○ |
| A3.3进行静脉血栓栓塞症防治对我的健康有好处 | ○ | ○ | ○ | ○ | ○ |

A4 感知健康行为的障碍

|  | 非常同意 | 同意 | 不确定 | 不同意 | 非常不同意 |
| --- | --- | --- | --- | --- | --- |
| A4.1即使进行了静脉血栓栓塞症防治，我仍担心会患上该疾病或病情加重 | ○ | ○ | ○ | ○ | ○ |
| A4.2我担心进行静脉血栓栓塞症防治会发生不良反应（如出血、下肢皮肤损伤等） | ○ | ○ | ○ | ○ | ○ |
| A4.3进行静脉血栓栓塞症防治要花一些时间 | ○ | ○ | ○ | ○ | ○ |
| A4.4进行静脉血栓栓塞症防治花钱较多 | ○ | ○ | ○ | ○ | ○ |

A5 自我效能

|  | 非常不同意 | 不同意 | 不确定 | 同意 | 非常同意 |
| --- | --- | --- | --- | --- | --- |
| A5.1我能主动了解静脉血栓栓塞症相关信息 | ○ | ○ | ○ | ○ | ○ |
| A5.2我能按照医护人员要求配合做好静脉血栓栓塞症的防治措施 | ○ | ○ | ○ | ○ | ○ |

A6 行动诱因

|  | 非常不同意 | 不同意 | 不确定 | 同意 | 非常同意 |
| --- | --- | --- | --- | --- | --- |
| A6.1医护人员向我解释静脉血栓栓塞症知识，让我更加注重防治该疾病 | ○ | ○ | ○ | ○ | ○ |
| A6.2其他人患有静脉血栓栓塞症的经历，让我更加注重防治该疾病 | ○ | ○ | ○ | ○ | ○ |
| A6.3从书刊杂志、电视、网络等途径得知的静脉血栓栓塞症知识，让我更加注重防治该疾病 | ○ | ○ | ○ | ○ | ○ |
| A6.4身体目前的状态提示我进行静脉血栓栓塞症的防治是非常有必要的 | ○ | ○ | ○ | ○ | ○ |

**Part 2: Questionnaire on Patients’ Attitudes Regarding the Prevention and Treatment of Venous Thromboembolism**

Please select the option that best matches your current thoughts.

**A1 Perceived Susceptibility to VTE**

|  | Strongly Disagree | Disagree | Uncertain | Agree | Strongly Agree |
| --- | --- | --- | --- | --- | --- |
| A1.1 I believe that regular assessment of thrombosis risk is necessary. | ○ | ○ | ○ | ○ | ○ |
| A1.2 I believe that thrombosis risk is dynamic. | ○ | ○ | ○ | ○ | ○ |
| A1.3 I believe that if new discomfort symptoms appear, or if existing symptoms worsen, I should see a doctor as soon as possible. | ○ | ○ | ○ | ○ | ○ |

**A2 Perceived Severity of VTE**

|  | Strongly Disagree | Disagree | Uncertain | Agree | Strongly Agree |
| --- | --- | --- | --- | --- | --- |
| A2.1 I believe that having venous thromboembolism will affect my health. | ○ | ○ | ○ | ○ | ○ |
| A2.2 I believe that having venous thromboembolism will cause psychological stress. | ○ | ○ | ○ | ○ | ○ |
| A2.3 I believe that having venous thromboembolism will affect my future work and life. | ○ | ○ | ○ | ○ | ○ |
| A2.4 I believe that having venous thromboembolism will impose a financial burden on my family. | ○ | ○ | ○ | ○ | ○ |

**A3 Perceived Benefits of Health Behaviors**

|  | Strongly Disagree | Disagree | Uncertain | Agree | Strongly Agree |
| --- | --- | --- | --- | --- | --- |
| A3.1 Preventing and treating venous thromboembolism can reduce the incidence of this disease and its complications. | ○ | ○ | ○ | ○ | ○ |
| A3.2 Preventing and treating venous thromboembolism can increase my sense of security. | ○ | ○ | ○ | ○ | ○ |
| A3.3 Preventing and treating venous thromboembolism is beneficial to my health. | ○ | ○ | ○ | ○ | ○ |

**A4 Perceived Barriers to Health Behaviors**

|  | Strongly Disagree | Disagree | Uncertain | Agree | Strongly Agree |
| --- | --- | --- | --- | --- | --- |
| A4.1 Even with VTE prevention and treatment, I am still worried about suffering from the disease or getting worse. | ○ | ○ | ○ | ○ | ○ |
| A4.2 I am concerned about the adverse reactions (such as bleeding, skin damage to the lower limbs, etc.) that may occur during the prevention and treatment of venous thromboembolism. | ○ | ○ | ○ | ○ | ○ |
| A4.3 Preventing venous thromboembolism takes time. | ○ | ○ | ○ | ○ | ○ |
| A4.4 Preventing venous thromboembolism costs a lot. | ○ | ○ | ○ | ○ | ○ |

**A5 Self-Efficacy**

|  | Strongly Disagree | Disagree | Uncertain | Agree | Strongly Agree |
| --- | --- | --- | --- | --- | --- |
| A5.1 I can proactively learn about venous thromboembolism. | ○ | ○ | ○ | ○ | ○ |
| A5.2 I can follow the instructions of the medical staff to prevent and treat venous thromboembolism. | ○ | ○ | ○ | ○ | ○ |

**A6 Cues to Action**

|  | Strongly Disagree | Disagree | Uncertain | Agree | Strongly Agree |
| --- | --- | --- | --- | --- | --- |
| A6.1 The medical staff informed me about venous thromboembolism, which makes me more aware of the need to fight this disease. | ○ | ○ | ○ | ○ | ○ |
| A6.2 The experiences of others with venous thromboembolism have made me more aware of the need to fight this disease. | ○ | ○ | ○ | ○ | ○ |
| A6.3 Information from books, magazines, television, and the internet about venous thromboembolism makes me more aware of the need to fight this disease. | ○ | ○ | ○ | ○ | ○ |
| A6.4 My current physical condition suggests that it is very necessary to prevent and treat venous thromboembolism. | ○ | ○ | ○ | ○ | ○ |

**第三部分 《患者静脉血栓栓塞症防治-行为问卷》**

请选择最符合您当前行为状态的选项

P1 优化抗栓管理

|  | 从不这样 | 很少这样 | 有时这样 | 经常这样 | 完全这样 |
| --- | --- | --- | --- | --- | --- |
| P1.1我会定期评估自己发生静脉血栓的风险 | ○ | ○ | ○ | ○ | ○ |
| P1.2我会主动和医生联系了解自己的血栓风险情况 | ○ | ○ | ○ | ○ | ○ |
| *P1.3-P1.4行机械预防者回答* | | | | | |
| P1.3我能遵医嘱按医生要求进行机械预防（梯度压力弹力袜、间歇充气加压装置、足底加压泵） | ○ | ○ | ○ | ○ | ○ |
| P1.4我会主动向医护人员反馈机械预防使用情况 | ○ | ○ | ○ | ○ | ○ |
| *P1.5-P1.7使用抗凝药物者回答* | | | | | |
| P1.5我能遵医嘱按医生要求的剂量使用抗凝药物（华法林、利伐沙班、艾多沙班、达比加群、低分子肝素、普通肝素等） | ○ | ○ | ○ | ○ | ○ |
| P1.6我能遵医嘱每天按时使用抗凝药物 | ○ | ○ | ○ | ○ | ○ |
| P1.7我能遵医嘱坚持使用抗凝药物直至疗程结束 | ○ | ○ | ○ | ○ | ○ |

P2出血风险管理

|  | 从不这样 | 很少这样 | 有时这样 | 经常这样 | 完全这样 |
| --- | --- | --- | --- | --- | --- |
| P2.1我会定期评估自己的出血风险 | ○ | ○ | ○ | ○ | ○ |
| P2.2我会主动和医生联系了解自己的出血风险情况 | ○ | ○ | ○ | ○ | ○ |
| P2.3我会主动监测各类出血现象（例如皮肤淤血、牙龈出血、眼球片状出血、鼻出血、血尿、血便等） | ○ | ○ | ○ | ○ | ○ |
| P2.4我会主动了解当前服用药物带来的出血风险 | ○ | ○ | ○ | ○ | ○ |

P3并发症监测

|  | 从不这样 | 很少这样 | 有时这样 | 经常这样 | 完全这样 |
| --- | --- | --- | --- | --- | --- |
| P3.1我能主动和医生联系反映自己的身体不适症状 | ○ | ○ | ○ | ○ | ○ |
| P3.2我能遵医嘱按时完成静脉血栓栓塞症相关的检查（检查项目包括超声、CT等影像学检查、心电图检查、肺功能检查、抽血化验等） | ○ | ○ | ○ | ○ | ○ |
| P3.3我能及时关注自己的各项检查结果（包括超声、CT等影像学检查、心电图检查、肺功能检查、抽血化验等） | ○ | ○ | ○ | ○ | ○ |

P4数字健康管理

|  | 从不这样 | 很少这样 | 有时这样 | 经常这样 | 完全这样 |
| --- | --- | --- | --- | --- | --- |
| P4.1我能主动使用智能手机、平板电脑中的健康应用程序来获取健康知识 | ○ | ○ | ○ | ○ | ○ |
| P4.2我能主动使用智能手环、智能手表、活动跟踪器和/或智能手机、平板电脑中的健康应用程序来监控健康状况 | ○ | ○ | ○ | ○ | ○ |
| P4.3我能主动使用智能手环、智能手表、活动跟踪器和/或智能手机、平板电脑中的健康应用程序来监控服药情况 | ○ | ○ | ○ | ○ | ○ |
| P4.4我能主动使用智能手机、平板电脑中的健康应用程序与医生进行网络互动来解答自己的健康问题 | ○ | ○ | ○ | ○ | ○ |

P5运动和康复

|  | 从不这样 | 很少这样 | 有时这样 | 经常这样 | 完全这样 |
| --- | --- | --- | --- | --- | --- |
| P5.1我能按计划进行规律的运动锻炼（如伸展运动、肢体关节活动、太极拳、骑自行车、广场舞、快步走、跑步等） | ○ | ○ | ○ | ○ | ○ |
| P5.2我能根据身体状况，动态地调整运动量 | ○ | ○ | ○ | ○ | ○ |
| P5.3我能避免坐矮板凳、久站、久坐或久蹲的行为 | ○ | ○ | ○ | ○ | ○ |

P6合并风险及疾病管理

|  | 从不这样 | 很少这样 | 有时这样 | 经常这样 | 完全这样 |
| --- | --- | --- | --- | --- | --- |
| P6.1我能定期主动监测自己的血压、心率 | ○ | ○ | ○ | ○ | ○ |
| P6.2我能定期主动监测自己的体重 | ○ | ○ | ○ | ○ | ○ |
| P6.3我能保证均衡的饮食(每餐有蔬菜、肉类和主食)，摄入富含纤维素的食物（如芹菜、粗粮等） | ○ | ○ | ○ | ○ | ○ |
| P6.4我能积极治疗所患的原发疾病，避免静脉血栓栓塞症的发生或病情加重 | ○ | ○ | ○ | ○ | ○ |
| P6.5我能以乐观向上的态度对待生活及康复过程 | ○ | ○ | ○ | ○ | ○ |

**Part 3: Behavior Questionnaire on the Prevention and Treatment of Venous Thromboembolism in Patients**

Please select the option that best describes your current behavior.

**P1 Appropriate Antithrombotic Management**

|  | Never | Rarely | Sometimes | Often | Always |
| --- | --- | --- | --- | --- | --- |
| P1.1 I am regularly assessing my risk of venous thrombosis. | ○ | ○ | ○ | ○ | ○ |
| P1.2 I will proactively contact my doctor to assess my risk of thrombosis. | ○ | ○ | ○ | ○ | ○ |
| *P1.3-P1.4 For those using mechanical prevention measures:* | | | | | |
| P1.3 I can follow the doctor's instructions and use mechanical prevention (compression stockings, intermittent pneumatic compression devices, foot pumps) as prescribed by the doctor. | ○ | ○ | ○ | ○ | ○ |
| P1.4 I will proactively feedback to medical staff on the use of mechanical prevention. | ○ | ○ | ○ | ○ | ○ |
| *P1.5-P1.7 For those using anticoagulant medications:* | | | | | |
| P1.5 I can adhere to the doctor's instructions to use anticoagulants (warfarin, rivaroxaban, edoxaban, dabigatran, low molecular weight heparin, unfractionated heparin, etc.) . | ○ | ○ | ○ | ○ | ○ |
| P1.6 I can take anticoagulants on time every day as prescribed. | ○ | ○ | ○ | ○ | ○ |
| P1.7 I can continue using anticoagulants until the end of the treatment course as prescribed. | ○ | ○ | ○ | ○ | ○ |

**P2 Bleeding Risk Management**

|  | Never | Rarely | Sometimes | Often | Always |
| --- | --- | --- | --- | --- | --- |
| P2.1 I regularly assess my risk of bleeding. | ○ | ○ | ○ | ○ | ○ |
| P2.2 I proactively contact my doctor to assess my risk of bleeding. | ○ | ○ | ○ | ○ | ○ |
| P2.3 I proactively monitor for signs of bleeding (e.g., bruising, gum bleeding, eye hemorrhage, nosebleeds, hematuria, blood in the stool). | ○ | ○ | ○ | ○ | ○ |
| P2.4 I proactively learn about the bleeding risks associated with my current medications. | ○ | ○ | ○ | ○ | ○ |

**P3 Complication Monitoring**

|  | Never | Rarely | Sometimes | Often | Always |
| --- | --- | --- | --- | --- | --- |
| P3.1 I can contact a doctor if I feel unwell. | ○ | ○ | ○ | ○ | ○ |
| P3.2 I can complete all necessary tests related to venous thromboembolism on time (e.g., ultrasound, CT imaging, ECG, pulmonary function tests, blood tests). | ○ | ○ | ○ | ○ | ○ |
| P3.3 I promptly pay attention to my test results (e.g., ultrasound, CT imaging, ECG, pulmonary function tests, blood tests). | ○ | ○ | ○ | ○ | ○ |

**P4 Digital Health Management**

|  | Never | Rarely | Sometimes | Often | Always |
| --- | --- | --- | --- | --- | --- |
| P4.1 I proactively use health applications on smartphones or tablets to obtain health knowledge. | ○ | ○ | ○ | ○ | ○ |
| P4.2 I proactively use smart bands, smart watches, activity trackers, and/or health applications on smartphones or tablets to monitor my health status. | ○ | ○ | ○ | ○ | ○ |
| P4.3 I proactively use smart bands, smart watches, activity trackers, and/or health applications on smartphones or tablets to monitor medication adherence. | ○ | ○ | ○ | ○ | ○ |
| P4.4 I proactively use health applications on smartphones or tablets to interact online with doctors to address my health concerns. | ○ | ○ | ○ | ○ | ○ |

**P5 Exercise and Rehabilitation**

|  | Never | Rarely | Sometimes | Often | Always |
| --- | --- | --- | --- | --- | --- |
| P5.1 I can perform regular exercise as planned (e.g., stretching, limb joint movements, Tai Chi, cycling, square dancing, brisk walking, running). | ○ | ○ | ○ | ○ | ○ |
| P5.2 I can adjust the amount of exercise dynamically according to my physical condition. | ○ | ○ | ○ | ○ | ○ |
| P5.3 I can avoid behaviors such as sitting on low stools, standing for long periods, sitting for long periods, or squatting for long periods. | ○ | ○ | ○ | ○ | ○ |

**P6 Facilitate The Management of Vascular Risks and Comorbidities**

|  | Never | Rarely | Sometimes | Often | Always |
| --- | --- | --- | --- | --- | --- |
| P6.1 I regularly monitor my blood pressure and heart rate. | ○ | ○ | ○ | ○ | ○ |
| P6.2 I regularly monitor my weight. | ○ | ○ | ○ | ○ | ○ |
| P6.3 I ensure a balanced diet (including vegetables, meat, and staple foods at every meal) and intake of fiber-rich foods (e.g., celery, whole grains). | ○ | ○ | ○ | ○ | ○ |
| P6.4 I actively treat the underlying disease to prevent the occurrence or aggravation of venous thromboembolism. | ○ | ○ | ○ | ○ | ○ |
| P6.5 I maintain an optimistic attitude towards life and the rehabilitation process. | ○ | ○ | ○ | ○ | ○ |

**Appendix B:** All items from the World Health Organisation Trial dataset

| **Data category** | **Information** |
| --- | --- |
| **Primary registry and trial identifying number** | NCT06350331 |
| **Date of registration in primary registry** | March 30, 2023 |
| **Secondary identifying numbers** | n/a |
| **Source of funding** | 14th Five-Year National Key Research and Development Program "Research on the Prevention and Treatment of Common and Frequently Occurring Diseases" Key Project |
| **Primary sponsor** | Sixth Medical Center of the Chinese People's Liberation Army General Hospital |
| **Secondary sponsor(s)** | n/a |
| **Contact for public queries** | dor_guoyt@hotmail.com |
| **Contact for scientific queries** | dor_guoyt@hotmail.com |
| **Public title** | SmaVTE |
| **Scientific title** | Smart Technology Facilitated Patient-centered Venous Thromboembolism Management: A Randomised Controlled Trial (The SmaVTE Study) |
| **Countries of recruitment** | China |
| **Health condition(s) or problem(s) studied** | Venous Thromboembolism (VTE) |
| **Intervention(s)** | mVTEA will assist in the management of patients during the post-hospitalization follow-up phase. The mVTEA's doctor terminal automatically sends VTE-related health education materials in different frequencies and contents based on the patient's knowledge of VTE prevention and treatment, as well as their risk of thrombosis and bleeding during follow-up. In addition, thrombosis physicians on the mVTEA's doctor terminal can deliver health education to patients based on their condition. This can be done through the mVTEA doctor-patient communication module, which includes text, photo, and voice interactions. |
| **Key inclusion and exclusion criteria** | Inclusion criteria:  Participants were considered eligible for inclusion in the study only if they met all of the following criteria：   - Inpatients ≥ 18 years of age at admission - Previous or current definitive diagnosis of deep vein thrombosis and/or pulmonary embolism by imaging, or at high risk of VTE at discharge: Padua score ≥4 for medical patients and Caprini score ≥5 for surgical patients - Signed informed consent   Exclusion criteria:   - Mental disorder or a combination of other serious diseases leading to incapacity for independent living - Inability to use smartphones, computer tablets, and other smart devices - Being pregnant or breastfeeding - Have participated in similar trials or are undergoing other clinical trials |
| **Study type** | Randomised controlled trial |
| **Date of first enrolment** | August 2024 |
| **Target sample size** | 256 participants |
| **Recruitment status** | Ongoing recruitment |
| **Primary outcome(s)** | Patients' knowledge, attitudes and practices (KAP) on VTE |
| **Key secondary outcomes** | - Scores on each domain of the KAP questionnaire on VTE - Quality of life - VTE events - Chronic thromboembolic pulmonary hypertension - Chronic thromboembolic pulmonary disease - Post-pulmonary embolism syndrome - Major bleeding events - VTE-related hospitalizations or rehospitalizations - Deaths - New-onset of atrial fibrillation or atrial flutter |

**Appendix C:** Informed Consent Form

知情同意告知页

**版本：V1.0/20240310**

尊敬的先生/女士：

您将被邀请参加一项科研课题的临床研究，请仔细阅读本知情同意书并慎重做出是否参加本项研究的决定。参加这项研究完全是您自主的选择。作为受试者，您必须在加入临床研究前给出您的书面同意书。当您的研究医生或者研究人员和您讨论知情同意书的时候，您可以让他/她给您解释您看不明白的地方。我们鼓励您在做出参与此项研究的决定之前，和您的家人及朋友进行充分讨论。您有权拒绝参加本研究，也可随时退出研究，且不会受到处罚，也不会失去您应有的权利。若您正在参加别的研究，请告知您的研究医生或者研究人员。本研究的背景、目的、研究过程及其他重要信息如下：

**1.研究背景**

静脉血栓栓塞症（VTE）包括深静脉血栓形成(DVT)和肺血栓栓塞症(PTE)，是造成伤残调整寿命年减少的主要原因，也是全球心血管疾病死亡的第三大原因，影响每年约1千万患者，且发病率随着年龄的增长而增加。静脉血栓栓塞症的防治和管理逐渐受到各学科的关注和重视，提高静脉血栓栓塞症规范预防率是国家医疗质量安全改进目标之一。然而，传统静脉血栓栓塞症医院就诊模式，存在救治成本高、优质医疗资源短缺、指南依从性欠佳、并发症未有效防治、诊后康复管理缺失等科学问题。近年来，智能技术（可穿戴设备、移动技术、人工智能等）正被探索应用于疾病及健康管理，在疾病预警、辅助决策支持、健康教育及健康维护显示其潜在的应用前景，有望解决血栓管理的学科瓶颈问题，实现优质医疗资源不同地域的可及性，完善血栓救治防支撑体系建设。

**2.研究名称和目的**

本研究来源于十四五国家重点研发计划“常见多发病防治研究”专项“肺血栓栓塞症综合防治体系构建及长期随访管理的系统化研究”项目（项目编号：2023YFC2507201）。本研究名称为“***智能技术辅助静脉血栓栓塞患者中心管理：单中心随机对照研究***”。

本研究目的旨在验证数字化血栓防治综合管理对患者血栓防治认知的影响（知信行调查问卷），进一步改善临床实践，为静脉血栓栓塞症人群管理提供科学依据。

**3.研究方法和内容**

本研究为单中心、随机对照研究。计划招募VTE高风险及VTE确诊患者，入组后按1:1比例随机分配至数字化血栓防治综合管理组及常规管理组。其中，数字化血栓防治综合管理组，将使用居家康复血栓应用程序进行数字化血栓防治综合管理；常规管理组，将按照当地临床实践，给予出院后常规管理，并记录管理情况，包括患者门诊就诊，抗凝药物使用，化验检查及再入院等。出院后完成至少3个月随访，以验证数字化血栓防治综合管理对患者血栓防治认知的影响（知信行调查问卷）。**本研究计划纳入256例受试者，每组128例。**

在您入选研究前，研究医生或者研究人员将询问、记录您的病史，并根据您入院前或入院后检查结果进行筛选是否符合研究入选标准。当您了解参加研究可能的获益和风险，自愿参加并签署经伦理委员会批准的知情同意书，经研究医生或者研究人员判断您符合入排标准后，您将在出院前填写《电子健康素养调查问卷》、《患者静脉血栓栓塞症防治数字健康教育调查问卷》，和《EQ-5D-5L欧洲五维健康量表》。

在您出院后第3个月，研究医生将对您进行临床随访，记录您出院后至随访时间点间，包括门诊就诊、药物使用、化验检查及再住院等医疗信息，了解并评估您的健康状况。同时，您将再次填写《患者静脉血栓栓塞症防治数字健康教育调查问卷》和《EQ-5D-5L欧洲五维健康量表》。

随访方式包括住院、门诊、电话、居家康复血栓应用程序（仅限采用数字化血栓防治综合管理的患者）等多种随访方式。

**4.研究入排标准**

4.1 入选标准：

只有符合以下所有条件的参与者才被视为有资格参与研究：

（1）入院时年龄≥18岁的住院患者；

（2）既往或当前经影像学检查明确诊断DVT和/或PTE，或出院时以下任一VTE风险评分为高危：内科患者Padua评分（≥4分）、外科患者Caprini评分（≥5分）；

（3）签署知情同意。

4.2 排除标准：

（1）神智障碍或合并其它严重疾病无自主生活能力；

（2）无法使用智能手机、电脑平板等智能设备；

（3）处于妊娠期或哺乳期；

（4）曾参加过类似试验或正在接受其他临床试验。

**5.研究过程和期限**

确定您可以参加本研究后，您在住院期间由您的主治医生制定的治疗方案，本研究不会进行任何干预。在您出院后，本研究将为您制定详细的随访计划。预期您参与研究的持续时间为3个月左右，主要为在您出院后第3个月进行临床随访。

**6.研究的资金来源和可能的利益冲突**

本研究的研究资金来源于十四五国家重点研发计划常见多发病防治研究专项，无相关的利益冲突。

**7.您可能的受益**

潜在受益：参与本研究后将为您制定详尽的随访计划，监测并发症风险及用药风险，有助于规范治疗方案，从而可能阻止/减缓疾病的进展。我们希望从您参与的本研究中得到的信息在将来能够使您或与您病情相同的病人获益。

**8.您可能的风险与不适**

本研究为观察性研究，不对治疗过程进行干预，因此参加本研究不会给您带来特定医疗风险。

本研究可能存在信息安全方面的风险，我们会尽全力保护您提供的信息不被泄露。本研究中我们所问您的一些问题可能会让您感到不舒服，你可以拒绝回答此类问题，同时，研究过程中您随时都可以休息。在研究中任何时刻，您都可以退出本研究。

**9.您与研究相关伤害的治疗和经济补偿**

因本研究不会对您产生伤害，因此不设置与研究相关伤害的治疗和经济补偿。

**10.您可能被分配到的研究组别**

本研究为随机对照研究，根据出院后随访管理方式的不同，按1:1比例随机分配至两个组别之一：（1）数字化血栓防治综合管理组；（2）常规管理组。因此，您有可能被分配至数字化血栓防治综合管理组，也有可能被分配至常规管理组。

**11.本研究之外的替代诊疗方法**

本研究为观察性研究，不对治疗过程进行干预。根据您的病情，除了参与本研究，您可以接受您的医生提供的常规治疗。由于本研究不涉及对您的治疗，故不存在替代治疗方法。

**12.医疗记录的保密方式**

我们深知个人信息对您的重要性，尤其在临床研究当中，我们尊重并保护您的隐私，并承诺会严格遵守法律法规要求且参考业界成熟的安全标准保护您的个人信息安全，且超过研究期限后不会以任何理由获取或传播您的个人隐私及信息。

在您和其他受试者的理解和协助下，通过本项目研究的结果可能会在医学杂志上发表，但是我们会按照法律的要求为您的研究记录保密。您的个人信息将受到严格保密，除非应相关法律要求，您的个人信息不会被泄露。必要时，政府管理部门和医院伦理委员会及其它相关研究人员可以按规定查阅您的资料。

**13.研究期间可能获得的免费诊疗项目和其他相关补助**

本研究为观察性研究，不对治疗过程进行干预，因此无研究所用的药物/器械及相关检查费用。对于您同时合并的其他疾病所需的常规治疗和检查，将不在免费的范围之内。无其他相关补助。

**14.自愿参加、退出研究**

您可以选择不参加本项研究，或者在任何时候通知研究者后退出研究而不会遭到歧视或报复，您的任何医疗待遇与权益不会因此而受到影响。

如果您需要其他诊断**/**治疗，或者您没有遵守研究方案，或者有任何其他合理原因，研究者可以终止您继续参与本项研究。

您可随时了解与本研究相关的信息资料和研究进展，如果您有与本研究有关的问题，或您在研究过程中发生了任何不适与损伤，或有关于本项研究参加者权益方面的问题，您可以通过 18518671467 与 金至赓 医生 联系，和/或通过 010-66957608 与解放军总医院第六医学中心医学伦理委员会办公室联系。

**知情同意签字页**

**受试者声明**

我已经仔细阅读了本知情同意书，我有机会提问而且所有问题均已得到解答。我理解参加本项研究是自愿的，我可以选择不参加本项研究，或者在任何时候通知研究者后退出而不会遭到歧视或报复，我的任何医疗待遇与权益不会因此而受到影响。

如果我需要其他诊断**/**治疗，或者我没有遵守研究方案，或者有其他合理原因，研究者可以终止我继续参与本项临床研究。

我自愿同意参加该项临床研究，我将收到一份签过字的“知情同意书”副本。

受试者签名： 日期： 年 月 日

受试者因无行为能力等原因不能签署知情同意书的，由其监护人签署。

监护人签名： 日期： 年 月 日

与受试者关系：

受试者不能签署知情同意书的理由：

**研究者声明**

我已准确地将知情同意书内容告知受试者并对受试者的提问进行了解答，受试者自愿参加本项临床研究。

研究者签名： 日期： 年 月 日

**Informed Consent Informational Page**

**Version: V1.0/20240310**

Dear Sir/Madam:

You are invited to participate in a clinical study of a research project. Please read this informed consent form carefully and make an informed decision about whether or not to participate in this study. Participation in this study is entirely voluntary. As a participant, you must give your written consent before you can participate in the clinical study. When your study investigator or study staff discusses the informed consent form with you, you can ask him/her to explain anything you do not understand. We encourage you to discuss the decision to participate in this study with your family and friends. You have the right to refuse to participate in this study, or to withdraw from the study at any time, without penalty or loss of any rights to which you are otherwise entitled. If you are already participating in another study, please inform your study investigator or researcher. The background, purpose, study procedures, and other important information about this study are listed below:

**1. Background**

Venous thromboembolism (VTE), which includes deep vein thrombosis (DVT) and pulmonary thromboembolism (PTE), is the leading cause of disability-adjusted life-years and the third leading cause of cardiovascular deaths globally, affecting approximately 10 million patients per year, with the incidence increasing with age. The prevention and management of venous thromboembolism have increasingly gained attention across various disciplines, and improving the standardized prevention rate of VTE is one of the national healthcare quality and safety improvement goals. However, the traditional hospital-based care model for venous thromboembolism presents several scientific challenges, including high treatment costs, a shortage of high-quality medical resources, poor adherence to clinical guidelines, ineffective prevention of complications, and a lack of post-discharge rehabilitation management. In recent years, smart technologies (such as wearable devices, mobile technologies, and artificial intelligence) are being explored for their application in disease and health management. These technologies show potential in areas such as disease surveillance, decision support, health education, and health maintenance, with the promise of addressing bottlenecks in thrombus management, ensuring the accessibility of high-quality medical resources across regions, and enhancing the infrastructure for thrombus prevention and treatment.

**2. Title and purpose of the study**

This study is conducted under the 14th Five-Year National Key Research and Development Program "Research on the Prevention and Treatment of Common and Frequently Occurring Diseases" Key Project (2023YFC2507201). The title of this study is ***Smart technology facilitated patient-centered venous thromboembolism management (SmaVTE): a randomised controlled trial***.

The aim of this study is to validate the effect of a smart technique-assisted patient-centered care mHealth app for managing VTE (mVTEA) on the knowledge, attitudes and practices (KAP) of VTE in patients with or at high risk of VTE, to further improve clinical practice, and to provide a scientific basis for the management of VTE.

**3. Research methodology**

This study is a single-center, randomized controlled study. It is planned to recruit patients with or at high risk of VTE, who will be randomly assigned to the mVTEA management group and routine management group in a 1:1 ratio after enrollment. In the mVTEA management group, patients will receive patient-centered VTE management facilitated by the mVTEA app after discharge. In the routine management group, the routine post-discharge management will be given in accordance with local clinical practice and the management will be documented, including patients' outpatient visits, anticoagulant medication use, laboratory tests, and readmission, etc. At least 3 months of follow-up will be completed after discharge to verify the effect of patient-centered VTE management facilitated by the mVTEA app on patients' KAP about VTE (VTE-KAP Questionnaire). **This study was planned to include 256 participants, 128 in each group.**

Before you are enrolled in the study, the study investigator or research staff will ask about and record your medical history and screen you for meeting the study enrollment criteria based on your pre-hospital or post-hospital test results. After you have been informed of the possible benefits and risks of participating in the study, you have voluntarily signed the informed consent form approved by the ethics committee, and the study investigator or researcher has determined that you meet the entry criteria, you should complete the EuroQol 5-Dimension 5-Level ( EQ-5D-5L), The Simplified Chinese version of the eHealth Literacy Scale (C-eHEALS), and KAP questionnaire on VTE at discharge.

During the 3rd month after your discharge from the hospital, the study investigator will conduct a clinical follow-up visit with you to record medical information, including outpatient visits, medication use, laboratory tests, and re-hospitalization, to learn about and assess your health status between your discharge and the time point of the follow-up visit. At the same time, you will fill out the EQ-5D-5L and KAP questionnaire on VTE.

Follow-up methods include hospitalization, outpatient visits, telephone calls, and the use of mVTEA app (only for patients in the mVTEA management group).

**4. Study inclusion and exclusion criteria**

4.1 Inclusion criteria:

Participants were considered eligible for inclusion in the study only if they met all of the following criteria：

1. Inpatients ≥ 18 years of age at admission
2. Previous or current definitive diagnosis of deep vein thrombosis and/or pulmonary embolism by imaging, or at high risk of VTE at discharge: Padua score ≥4 for medical patients and Caprini score ≥5 for surgical patients
3. Signed informed consent

4.2 Exclusion criteria:

1. Mental disorder or a combination of other serious diseases leading to incapacity for independent living
2. Inability to use smartphones, computer tablets, and other smart devices
3. Being pregnant or breastfeeding
4. Have participated in similar trials or are undergoing other clinical trials

**5. Research process and duration**

After you have been confirmed as eligible for the study, your doctor will develop a treatment plan for you during your hospital stay. The study will not intervene in any way. After you are discharged from the hospital, the study will develop a detailed follow-up plan for you. The expected duration of your participation in the study is about 3 months, mainly for clinical follow-up in the third month after your discharge from the hospital.

**6. Funding of research and possible conflicts of interest**

The research funding for this study was obtained from the 14th Five-Year National Key Research and Development Program "Research on the Prevention and Treatment of Common and Frequently Occurring Diseases" Key Project, and there is no relevant conflict of interest.

**7. Your potential benefits**

Potential benefits: Participation in this study will provide you with a detailed follow-up plan, monitor the risk of complications and medication risks, help standardize treatment plans, and may prevent/slow disease progression. We hope that the information we gain from you participating in this study will benefit you or patients with the same condition in the future.

**8. Your possible risks and discomforts**

This is an observational study and does not intervene in the course of treatment. Therefore, participation in this study does not pose a specific medical risk to you.

There may be information security risks associated with this study, and we will do our best to protect the information you provide from disclosure. Some of the questions we ask you in this study may make you uncomfortable and you can decline to answer such questions, and you can take breaks at any time during the study. You can withdraw from this study at any point during the study.

**9. Treatment and financial compensation for your research-related injuries**

Because this study will not result in harm to you, there is no treatment or financial compensation for research-related injuries.

**10. The research group to which you may be assigned**

This is a randomized controlled study in which you will be randomly assigned to one of two groups in a 1:1 ratio, depending on the type of post-discharge management: (1) the mVTEA management group; and (2) the routine management group. Therefore, you may be assigned to the mVTEA management group or to the routine management group.

**11. Alternative treatments to the present study**

This is an observational study with no intervention in the course of treatment. Depending on your condition, in addition to participating in this study, you may receive the usual care provided by your doctor. Since this study does not involve treatment for you, there are no alternative treatments.

**12. Confidentiality of medical records**

We understand the importance of your personal information, especially in clinical research, and we respect and protect your privacy. We are committed to strictly complying with legal and regulatory requirements and taking into account the industry's established security standards to protect the security of your personal information, and will not access or disseminate your personal information for any reason beyond the duration of the study.

The results of this project may be published in medical journals with your understanding and that of the other participants, but we will keep your research records confidential as required by law. Your personal information will be kept strictly confidential and will not be disclosed unless required by relevant laws. If necessary, government authorities, hospital ethics committees and other relevant researchers may access your data as required.

**13. Free medical services and other related benefits that may be obtained during the study period**

This study is an observational study that does not intervene in the course of treatment. Therefore, the costs of the drugs/devices used in the study and the associated examinations are not covered. The usual treatment and examinations for other diseases you may have at the same time are not covered. There are no other benefits.

**14. Voluntary participation, withdrawal from research**

You may choose not to participate in this study or to withdraw from the study at any time without discrimination or retaliation by notifying the investigator, and any of your medical treatment and rights will not be affected as a result.

The investigator may terminate you from the study if you require additional diagnostic/therapeutic procedures, if you do not comply with the study protocol, or for any other reasonable cause.

You have the right to access information and progress of the study at any time. If you have any questions about the study, or if you experience any discomfort or injury during the study, or if you have any questions about the rights and interests of the participants in this study, you can contact Dr. Jin Zhigeng at 18518671467 and/or the Medical Ethics Committee Office of the Sixth Medical Center of the PLA General Hospital at 010-66957608.

**Informed Consent Signature Page**

**Participant statement**

I have read this informed consent form carefully, I have had the opportunity to ask questions and all questions have been answered. I understand that participation in this study is voluntary and that I may choose not to participate in this study or withdraw at any time by notifying the investigator without discrimination or retaliation, and that none of my medical treatment or rights will be affected as a result.

The investigator may terminate my continued participation in this clinical study if I need other diagnosis/treatment, or if I fail to comply with the study protocol, or for any other valid reason.

I voluntarily agree to participate in this clinical study and will receive a copy of the signed Informed Consent Form.

Signature of the participant: Date:

If the participant is unable to sign the informed consent form due to incapacity, his/her guardian will sign it.

Signature of guardian: Date

Relationships with participant:

Reasons why participant could not sign the informed consent form:

**Investigator's statement**

I have accurately informed the subject of the contents of the informed consent form and answered the subject's questions, and the subject has volunteered to participate in this clinical study.

Signature of investigator: Date

**Appendix D:** Reporting checklist for protocol of a clinical trial based on the SPIRIT guidelines

| Section/item | Item No | Description | Addressed on page number |
| --- | --- | --- | --- |
| **Administrative information** | | |  |
| Title | 1 | Descriptive title identifying the study design, population, interventions, and, if applicable, trial acronym | 1 |
| Trial registration | 2a | Trial identifier and registry name. If not yet registered, name of intended registry | 4 |
|  | 2b | All items from the World Health Organization Trial Registration Data Set | Appendix B |
| Protocol version | 3 | Date and version identifier | 4 |
| Funding | 4 | Sources and types of financial, material, and other support | 13 |
| Roles and responsibilities | 5a | Names, affiliations, and roles of protocol contributors | 1,13 |
|  | 5b | Name and contact information for the trial sponsor | 13 |
|  | 5c | Role of study sponsor and funders, if any, in study design; collection, management, analysis, and interpretation of data; writing of the report; and the decision to submit the report for publication, including whether they will have ultimate authority over any of these activities | n/a |
|  | 5d | Composition, roles, and responsibilities of the coordinating centre, steering committee, endpoint adjudication committee, data management team, and other individuals or groups overseeing the trial, if applicable (see Item 21a for data monitoring committee) | n/a |
| Introduction |  |  |  |
| Background and rationale | 6a | Description of research question and justification for undertaking the trial, including summary of relevant studies (published and unpublished) examining benefits and harms for each intervention | 3-4 |
|  | 6b | Explanation for choice of comparators | 3-4 |
| Objectives | 7 | Specific objectives or hypotheses | 4 |
| Trial design | 8 | Description of trial design including type of trial (eg, parallel group, crossover, factorial, single group), allocation ratio, and framework (eg, superiority, equivalence, noninferiority, exploratory) | 4 |
| Methods: Participants, interventions, and outcomes | | |  |
| Study setting | 9 | Description of study settings (eg, community clinic, academic hospital) and list of countries where data will be collected. Reference to where list of study sites can be obtained | 4 |
| Eligibility criteria | 10 | Inclusion and exclusion criteria for participants. If applicable, eligibility criteria for study centres and individuals who will perform the interventions (eg, surgeons, psychotherapists) | 5,Table 1 |
| Interventions | 11a | Interventions for each group with sufficient detail to allow replication, including how and when they will be administered | 6-8 |
|  | 11b | Criteria for discontinuing or modifying allocated interventions for a given trial participant (eg, drug dose change in response to harms, participant request, or improving/worsening disease) | n/a |
|  | 11c | Strategies to improve adherence to intervention protocols, and any procedures for monitoring adherence (eg, drug tablet return, laboratory tests) | 6-8 |
|  | 11d | Relevant concomitant care and interventions that are permitted or prohibited during the trial | n/a |
| Outcomes | 12 | Primary, secondary, and other outcomes, including the specific measurement variable (eg, systolic blood pressure), analysis metric (eg, change from baseline, final value, time to event), method of aggregation (eg, median, proportion), and time point for each outcome. Explanation of the clinical relevance of chosen efficacy and harm outcomes is strongly recommended | 8-9 |
| Participant timeline | 13 | Time schedule of enrolment, interventions (including any run-ins and washouts), assessments, and visits for participants. A schematic diagram is highly recommended (see Figure) | 9,Table 5 |
| Sample size | 14 | Estimated number of participants needed to achieve study objectives and how it was determined, including clinical and statistical assumptions supporting any sample size calculations | 10 |
| Recruitment | 15 | Strategies for achieving adequate participant enrolment to reach target sample size | 4-5 |
| **Methods: Assignment of interventions (for controlled trials)** | | |  |
| Allocation: |  |  |  |
| Sequence generation | 16a | Method of generating the allocation sequence (eg, computer-generated random numbers), and list of any factors for stratification. To reduce predictability of a random sequence, details of any planned restriction (eg, blocking) should be provided in a separate document that is unavailable to those who enrol participants or assign interventions | 5 |
| Allocation concealment mechanism | 16b | Mechanism of implementing the allocation sequence (eg, central telephone; sequentially numbered, opaque, sealed envelopes), describing any steps to conceal the sequence until interventions are assigned | 5 |
| Implementation | 16c | Who will generate the allocation sequence, who will enrol participants, and who will assign participants to interventions | 5 |
| Blinding (masking) | 17a | Who will be blinded after assignment to interventions (eg, trial participants, care providers, outcome assessors, data analysts), and how | 5 |
|  | 17b | If blinded, circumstances under which unblinding is permissible, and procedure for revealing a participant’s allocated intervention during the trial | n/a |
| **Methods: Data collection, management, and analysis** | | |  |
| Data collection methods | 18a | Plans for assessment and collection of outcome, baseline, and other trial data, including any related processes to promote data quality (eg, duplicate measurements, training of assessors) and a description of study instruments (eg, questionnaires, laboratory tests) along with their reliability and validity, if known. Reference to where data collection forms can be found, if not in the protocol | 9 |
|  | 18b | Plans to promote participant retention and complete follow-up, including list of any outcome data to be collected for participants who discontinue or deviate from intervention protocols | 9 |
| Data management | 19 | Plans for data entry, coding, security, and storage, including any related processes to promote data quality (eg, double data entry; range checks for data values). Reference to where details of data management procedures can be found, if not in the protocol | 9 |
| Statistical methods | 20a | Statistical methods for analysing primary and secondary outcomes. Reference to where other details of the statistical analysis plan can be found, if not in the protocol | 10-11 |
|  | 20b | Methods for any additional analyses (eg, subgroup and adjusted analyses) | 10-11 |
|  | 20c | Definition of analysis population relating to protocol non-adherence (eg, as randomised analysis), and any statistical methods to handle missing data (eg, multiple imputation) | 10 |
| **Methods: Monitoring** | | |  |
| Data monitoring | 21a | Composition of data monitoring committee (DMC); summary of its role and reporting structure; statement of whether it is independent from the sponsor and competing interests; and reference to where further details about its charter can be found, if not in the protocol. Alternatively, an explanation of why a DMC is not needed | 9 |
|  | 21b | Description of any interim analyses and stopping guidelines, including who will have access to these interim results and make the final decision to terminate the trial | n/a |
| Harms | 22 | Plans for collecting, assessing, reporting, and managing solicited and spontaneously reported adverse events and other unintended effects of trial interventions or trial conduct | 9 |
| Auditing | 23 | Frequency and procedures for auditing trial conduct, if any, and whether the process will be independent from investigators and the sponsor | 9 |
| Ethics and dissemination | | |  |
| Research ethics approval | 24 | Plans for seeking research ethics committee/institutional review board (REC/IRB) approval | 11 |
| Protocol amendments | 25 | Plans for communicating important protocol modifications (eg, changes to eligibility criteria, outcomes, analyses) to relevant parties (eg, investigators, REC/IRBs, trial participants, trial registries, journals, regulators) | n/a |
| Consent or assent | 26a | Who will obtain informed consent or assent from potential trial participants or authorised surrogates, and how (see Item 32) | 5 |
|  | 26b | Additional consent provisions for collection and use of participant data and biological specimens in ancillary studies, if applicable | n/a |
| Confidentiality | 27 | How personal information about potential and enrolled participants will be collected, shared, and maintained in order to protect confidentiality before, during, and after the trial | 9 |
| Declaration of interests | 28 | Financial and other competing interests for principal investigators for the overall trial and each study site | 13 |
| Access to data | 29 | Statement of who will have access to the final trial dataset, and disclosure of contractual agreements that limit such access for investigators | 13 |
| Ancillary and post-trial care | 30 | Provisions, if any, for ancillary and post-trial care, and for compensation to those who suffer harm from trial participation | n/a |
| Dissemination policy | 31a | Plans for investigators and sponsor to communicate trial results to participants, healthcare professionals, the public, and other relevant groups (eg, via publication, reporting in results databases, or other data sharing arrangements), including any publication restrictions | 12 |
|  | 31b | Authorship eligibility guidelines and any intended use of professional writers | n/a |
|  | 31c | Plans, if any, for granting public access to the full protocol, participant-level dataset, and statistical code | 13 |
| Appendices |  |  |  |
| Informed consent materials | 32 | Model consent form and other related documentation given to participants and authorised surrogates | Appendix C |
| Biological specimens | 33 | Plans for collection, laboratory evaluation, and storage of biological specimens for genetic or molecular analysis in the current trial and for future use in ancillary studies, if applicable | n/a |
